# The Number and Type of Chaperone-Usher Fimbriae Reflect Phylogenetic Clade Rather than Host Range in Salmonella

**DOI:** 10.1128/msystems.00115-22

**Published:** 2022-04-25

**Authors:** Rachel A. Cheng, Renato H. Orsi, Martin Wiedmann

**Affiliations:** a Department of Food Science, Cornell University, Ithaca, New York, USA; Teagasc Food Research Centre

**Keywords:** *Salmonella*, chaperone-usher, fimbriae, host adaptation

## Abstract

Salmonella is one of the most successful foodborne pathogens worldwide, owing in part to its ability to colonize or infect a wide range of hosts. Salmonella serovars are known to encode a variety of different fimbriae (hairlike organelles that facilitate binding to surfaces); however, the distribution, number, and sequence diversity of fimbriae encoded across different lineages of Salmonella were unknown. We queried whole-genome sequence (WGS) data for 242 Salmonella enterica subsp. *enterica* (subspecies *enterica*) isolates from the top 217 serovars associated with isolation from humans and agricultural animals; this effort identified 2,894 chaperone-usher (CU)-type fimbrial usher sequences, representing the most conserved component of CU fimbriae. On average, isolates encoded 12 different CU fimbrial ushers (6 to 18 per genome), although the distribution varied significantly (*P* = 1.328E−08) by phylogenetic clade, with isolates in section Typhi having significantly fewer fimbrial ushers than isolates in clade A2 (medians = 10 and 12 ushers, respectively). Characterization of fimbriae in additional non-*enterica* subspecies genomes suggested that 8 fimbrial ushers were classified as being unique to subspecies *enterica* isolates, suggesting that the majority of fimbriae were most likely acquired prior to the divergence of subspecies *enterica*. Characterization of mobile elements suggested that plasmids represent an important vehicle facilitating the acquisition of a wide range of fimbrial ushers, particularly for the acquisition of fimbriae from other Gram-negative genera. Overall, our results suggest that differences in the number and type of fimbriae encoded most likely reflect differences in phylogenetic clade rather than differences in host range.

**IMPORTANCE** Fimbriae of the CU assembly pathway represent important organelles that mediate Salmonella’s interactions with host tissues and abiotic surfaces. Our analyses provide a comprehensive overview of the diversity of CU fimbriae in Salmonella spp., highlighting that the majority of CU fimbriae are distributed broadly across multiple subspecies and suggesting that acquisition most likely occurred prior to the divergence of subspecies *enterica*. Our data also suggest that plasmids represent the primary vehicles facilitating the horizontal transfer of diverse CU fimbriae in Salmonella. Finally, the observed high sequence similarity between some ushers suggests that different names may have been assigned to closely related fimbrial ushers that likely should be represented by a single designation. This highlights the need to establish standard criteria for fimbria classification and nomenclature, which will also facilitate future studies seeking to associate virulence factors with adaptation to or differences in the likelihood of causing disease in a given host.

## INTRODUCTION

Salmonella enterica is a leading cause of bacterial foodborne illness in the United States (1) and remains an important cause of diarrheal illness worldwide (2, 3). S. enterica is an appreciably diverse species, including 6 recognized subspecies (*enterica* [I], *salamae* [II], *arizonae* [IIIa], *diarizonae* [IIIb], *houtenae* [IV], and *indica* [VI]) (4), although recent studies suggest the presence of additional subspecies such as VII (5). Importantly, the majority of clinical infections are attributed to S. enterica subsp. *enterica* (subspecies *enterica*) serovars, although serovars from all lineages are capable of causing illness in humans (6). Salmonella isolates have historically been differentiated based on their somatic (O) and flagellar (H) antigens (7), although the number of recognized so-called “polyphyletic” serovars (i.e., clades of isolates with the same antigenic formula that do not share a most recent common ancestor [MRCA] with that same antigenic formula) is increasing, as reflected by increases in the number of whole-genome sequence (WGS) characterizations (8, 9). Not surprisingly, recent WGS characterizations have further identified several potentially novel subspecies (10, 11) and major phylogenetic clades within subspecies *enterica* (8). Subspecies *enterica* currently contains four major phylogenetic clades, A, B, C, and D (8); clade A may be further subclassified into clades A1 and A2 and section Typhi. Section Typhi includes Salmonella serovars Typhi and Paratyphi A, while clades A1 and A2 include Salmonella serovars Agona and Typhimurium, respectively. Finally, clades C and D contain serovars (e.g., Salmonella serovars Denver and Brazzaville, respectively) that are rarely associated with human clinical disease (8).

While most subspecies *enterica* serovars are considered host generalists, meaning that they are capable of colonizing and/or infecting a broad range of hosts, some cause disease in only one host (host-restricted serovars), and others are frequently associated with one host but may also cause disease in other hosts (host-adapted serovars). Host-restricted serovars include *S.* Typhi, *S.* Paratyphi A, and *S.* Sendai, which are considered host restricted to humans (12), while *S.* Gallinarum is host restricted to galliform birds (13). Examples of host-adapted serovars include *S.* Dublin to cattle (13) and *S.* Choleraesuis to pigs (14). Recent genomic analyses have also suggested that within some serovars, isolates cluster phylogenetically by host (15–17). However, host associations for most serovars remain largely unknown.

Salmonella’s interactions with host mucosal surfaces, such as those in the small intestine, are proposed to be primarily mediated by long, proteinaceous structures called fimbriae (18, 19). Previous studies have identified a role for fimbriae in facilitating interactions with specific cell types (20–22) as well as specific hosts (23, 24). Fimbriae are categorized based on their morphology, assembly pathway, or function, with the majority of fimbriae belonging to the chaperone-usher (CU) family. The assembly of CU fimbriae occurs when one or more chaperones transport fimbrial subunits to a membrane-embedded usher protein for translocation across the outer membrane; some CU fimbriae may also include a minor subunit known as the tip adhesin, the most distal subunit that mediates interactions with various surfaces. In 2007, Nuccio and Bäumler introduced the current organization of CU fimbriae into families based on the sequence identity of the usher subunit (18). These families are comprised of (i) alternative fimbriae, or α-fimbriae; (ii) classical fimbriae (β-, γ-, κ-, and π-fimbriae); and (iii) archaic fimbriae, or σ-fimbriae (18). Salmonella serovars are known to encode a variety of different fimbriae, although most are poorly expressed under standard laboratory culture conditions, and therefore, the binding target of most fimbriae expressed by Salmonella is unknown (25). Previous studies have reported that subspecies *enterica* serovars encode 5 to 14 different fimbriae (26–28). Many of the fimbrial gene clusters have been identified from WGS characterizations (18), although no standard criteria have been proposed for defining potentially novel fimbriae, and as such, at least 36 CU fimbriae have been described in Salmonella (25–27, 29–31).

Despite ongoing efforts to understand the roles of different CU fimbriae, the majority of studies have been limited to examining CU fimbriae in select serovars such as *S.* Typhimurium and *S.* Typhi, and therefore, the true diversity of fimbriae encoded by different lineages of Salmonella may be underestimated. Therefore, the goals of this study were to (i) identify and characterize the diversity of CU fimbrial ushers, representing the most conserved component of the CU fimbriae (18), among representative S. enterica subsp. *enterica* isolates commonly isolated from human and animal sources; (ii) perform additional characterization of CU fimbrial usher presence/absence among non-*enterica* subspecies isolates, which are generally rare among human and animal clinical isolates; and (iii) assess the distribution of intact and hypothetically disrupted coding sequences of fimbrial genes. These efforts allowed us to assess the CU fimbrial diversity in Salmonella as well as the acquisition and loss events that shaped the fimbriome of subspecies *enterica*, which may impact host range and host specificity.

## RESULTS

### The numbers of CU fimbrial ushers differ significantly by phylogenetic clade for subspecies *enterica* serovars.

**FIG 1:**
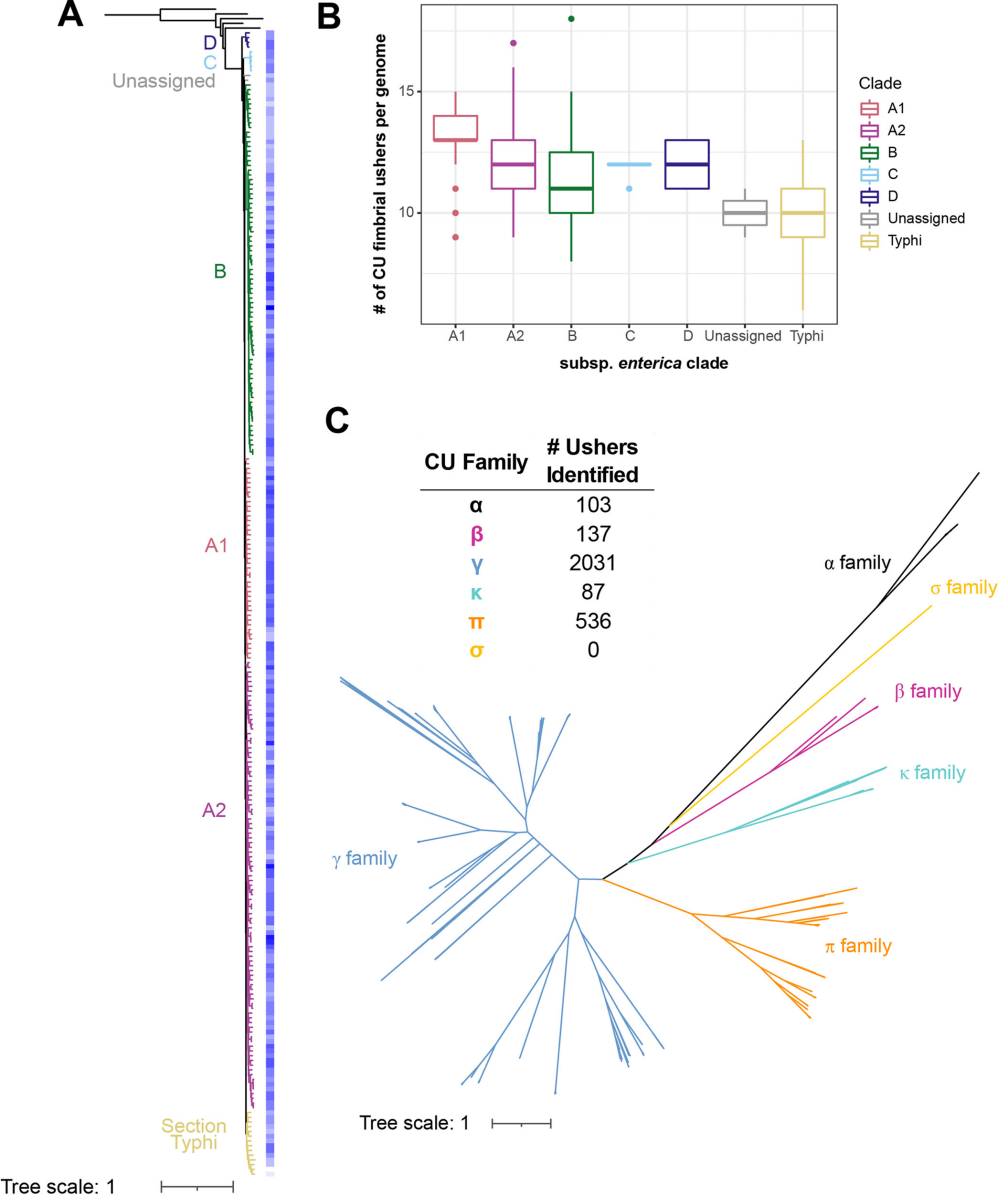
Overview of the Salmonella enterica subsp. *enterica* CU fimbriome. (A) Maximum likelihood phylogeny inferred from 13,926 core SNPs for 242 S. enterica subsp. *enterica* isolates (representing 217 unique serovars) and isolates representing 5 additional S. enterica subspecies. S. enterica subsp. *arizonae* was used to root the phylogeny (8, 30). Branches are colored by phylogenetic clade, which were defined to maintain consistency with those described previously (8). Rectangles shown exterior to the phylogeny are shaded to represent the number of CU fimbrial usher proteins detected per genome; darker colors represent higher numbers of fimbrial ushers detected (range, 6 to 18 fimbrial ushers per genome). Bootstrap values represent the percentage of times that the clustering observed in the phylogenetic tree was reproduced among the 1,000 ultrafast (UF) bootstrap repetitions. (B) Box-and-whisker plot summarizing the number of CU fimbrial usher protein-encoding genes (for all serovars shown in panel A) per S. enterica subsp. *enterica* clade. “Unassigned” designates isolates representing Salmonella serovars Poano and Lattenkamp, which could not be assigned to a phylogenetic clade. (C) Unrooted phylogeny for 2,894 CU fimbrial usher proteins identified among the 242 isolates in panel A and 30 reference CU fimbrial usher proteins. Clades are shaded to represent the CU family to which the usher proteins belong. Phylogeny was inferred using the LG+F+R7 model (selected based on the Bayesian information criterion [BIC] score).

To provide an updated view of the distribution of CU fimbriae among subspecies *enterica* serovars, representing the majority of serovars associated with clinical disease in humans and agricultural animals, we first curated a database that included WGS assemblies that represented serovars that (i) were prevalent among reported human clinical infections in the United States (6), (ii) were prevalent among nonhuman clinical or nonclinical isolates (32), and (iii) represented the major phylogenetic clades of subspecies *enterica* (8); for serovars that were known to be poly- or paraphyletic, multiple isolates were included to better capture the diversity of isolates within the serovar (see Materials and Methods for additional details about isolate selection). In total, our analyses included 242 isolates, representing 217 unique subspecies *enterica* serovars (Fig. 1A).

Our initial analyses using BLAST-based searches were unable to extract the entire coding sequences for divergent copies (i.e., amino acid sequences sharing low percent identities [%ID] with reference usher sequences) of select CU fimbrial usher proteins. Therefore, we instead used the InterPro database to annotate coding sequences from WGS data and extracted entries labeled “IPR000015” or “IPR035224,” representing the terms associated with usher and TcfC-like domains, respectively. Using this approach, we identified a total of 2,894 putative fimbrial usher proteins. On average, subspecies *enterica* serovars encoded 12 fimbrial usher proteins, ranging from 6 fimbrial usher proteins in *S.* Napoli (section Typhi) to 18 in *S.* Florida (clade B) (Fig. 1A). The number of CU fimbrial usher proteins encoded varied significantly by subspecies *enterica* phylogenetic clade (*P* = 1.328E−08 by a Kruskal-Wallis test), with isolates representing serovars in section Typhi (mean = 10 fimbrial ushers/genome) encoding significantly fewer fimbrial usher proteins than isolates representing serovars in clades A1 and A2 (means = 13 and 12 fimbrial ushers/genome, respectively) (Fig. 1B). Isolates representing serovars in clade A1 encoded significantly higher numbers of fimbrial ushers per genome than isolates representing serovars in clades A2, B, and C and section Typhi (*P* < 0.05 for all pairwise comparisons by a Wilcoxon rank sum test).

CU fimbrial usher proteins are categorized phylogenetically into families representing alternate (α-fimbriae), classical (β-, γ-, κ-, and π-fimbriae), or archaic (σ-fimbriae) fimbrial ushers (18). Not surprisingly, γ-fimbriae accounted for 70% of the CU fimbrial ushers identified (Fig. 1C). The second most numerous family was the π-fimbriae (19%), followed by the β-fimbriae (5%), α-fimbriae (4%), and κ-fimbriae (3%). None of the subspecies *enterica* isolates examined here encoded σ-fimbrial ushers.

Together, these results suggest that although the numbers of CU fimbrial ushers differ significantly by subspecies *enterica* clade, isolates encode an average of 12 CU fimbriae, with most belonging to the γ-fimbrial family.

### The α-fimbrial usher Tcf is encoded by multiple subspecies *enterica* serovars across all major phylogenetic clades except for clade C.

**FIG 2:**
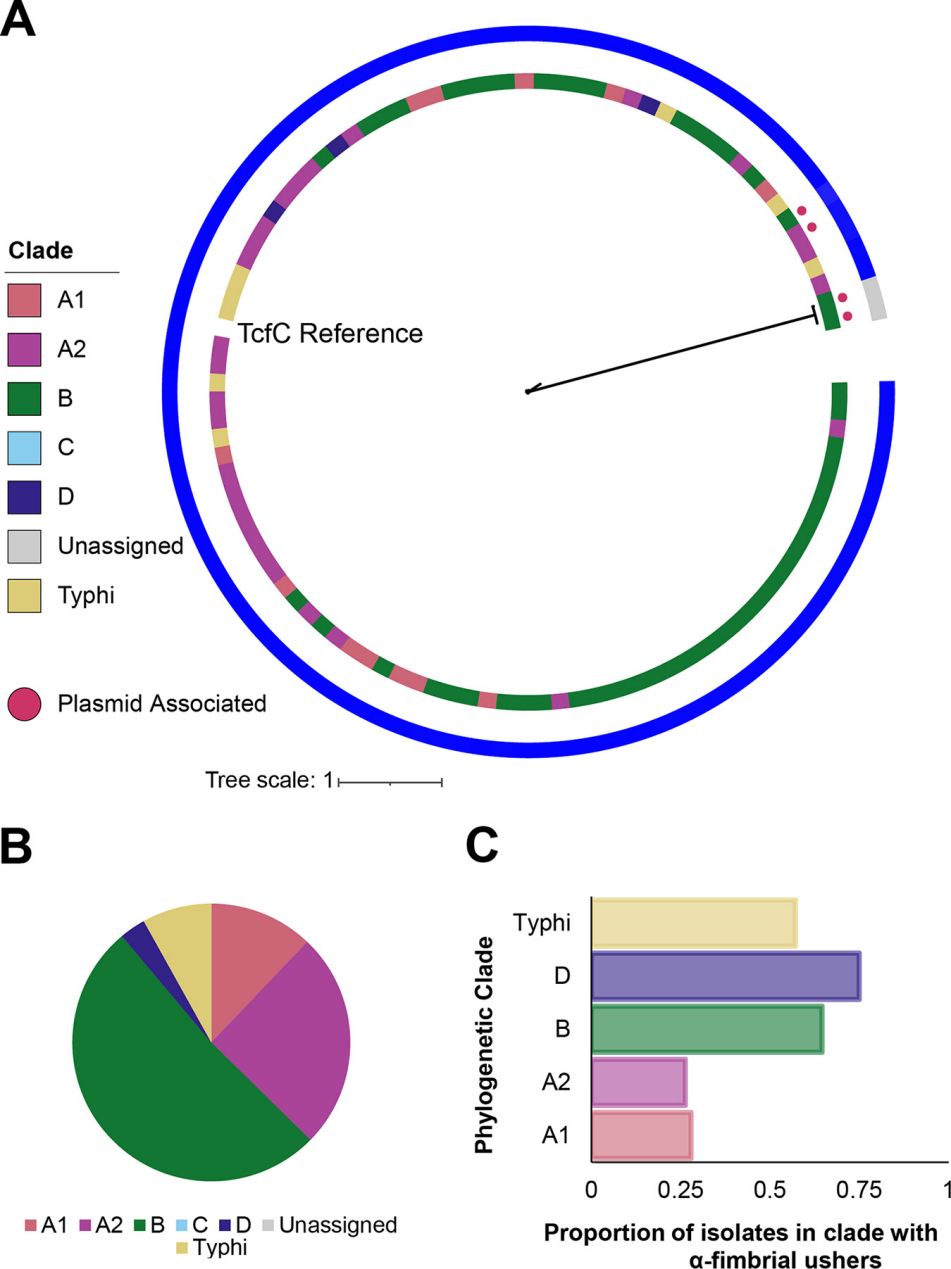
Distribution of genes encoding α-fimbrial ushers. (A) Phylogeny inferred from amino acid sequences for 103 coding sequences annotated as “IPR035224” (representing the TcfC-like domain). TcfC from *S.* Typhi CT18 is included as a reference. Bootstrap values represent the percentage of times that the clustering observed in the phylogenetic tree was reproduced among the 1,000 UF bootstrap repetitions. The innermost colored strip represents the phylogenetic clade of the isolate from which the usher was extracted. Magenta circles represent usher sequences that were detected on contigs associated with plasmids. The outermost colored strip represents the percent amino acid sequence identity shared between the usher and the TcfC reference; percent identity ranged from 29% (gray) to 100% (blue). Phylogeny was inferred using the FLU+F+R2 model (selected based on the BIC score). (B) Pie diagram displaying the proportion of the 103 α-fimbrial usher sequences as a function of the phylogenetic clade of the isolate in which they were identified. “Unassigned” includes isolates representing Salmonella serovars Poano and Lattenkamp, which could not be assigned to a major phylogenetic clade. (C) Bar chart displaying the proportion of isolates in each phylogenetic clade in which TcfC was detected. Four isolates had two copies of TcfC; duplicate sequences were removed so that each isolate was counted only once. Clade C and the two serovars that could not be placed into an existing clade (“Unassigned”) did not have any isolates with TcfC detected, and thus, these are excluded from the bar chart.

The Typhi colonization factor (Tcf) was the only α-fimbria previously identified among Salmonella isolates (26). A total of 103 CU α-fimbrial ushers identified here were annotated as IPR035224, which represents the InterPro Tcf-like motif. While 101 of these 103 CU α-fimbrial ushers shared ≥89% amino acid identity with the TcfC reference sequence from *S.* Typhi CT18, 2 sequences from isolates representing clade B Salmonella serovars Horsham and Roodepoort were highly divergent, sharing just 29% identity with the TcfC reference sequence but 99% identity with each other (Fig. 2A). These two divergent fimbrial ushers were both located on contigs associated with plasmids; BLAST searches identified a strain of Edwardsiella tarda that carried a homolog of this divergent α-fimbrial usher (98% coverage and 62% identity) (see Data Set S1 in the supplemental material), suggesting that this usher was likely horizontally acquired by *S.* Horsham and *S.* Roodepoort from a different bacterial genus such as *Edwardsiella*. For isolates representing clade A2 Salmonella serovars Bournemouth and Wandsworth, 2 distinct TcfC sequences were detected, suggesting that these isolates encode multiple copies of TcfC (Fig. 2A). For the isolate representing *S.* Wandsworth, one of the TcfC sequences was detected on a contig associated with a plasmid; while we were unable to confirm the presence of TcfC on a plasmid in the isolate representing *S.* Bournemouth, both TcfC sequences clustered together and shared higher percent sequence identity with each other (99% identity) than with the other, non-plasmid-associated TcfC encoded by the same strain (95% identity). This suggests that these isolates representing *S.* Bournemouth and *S.* Wandsworth likely encode chromosomal and plasmid copies of TcfC.

10.1128/msystems.00115-22.6 DATA SET S1 Annotations for additional fimbrial gene cluster genes for divergent ushers and tBLASTn results for divergent ushers. Download Data Set S1, XLSX file, 0.1 MB. Copyright © 2022 Cheng et al. 2022 Cheng et al. https://creativecommons.org/licenses/by/4.0/ This content is distributed under the terms of the Creative Commons Attribution 4.0 International license.

Although the Tcf fimbria was first identified in *S.* Typhi, we detected TcfC among isolates representing serovars in all clades except for clade C and the two serovars that could not be assigned to a clade (Fig. 1A and Fig. 2B). While 57% of isolates representing section Typhi serovars had TcfC, serovars in clades D and B had higher proportions of TcfC-positive serovars (75% and 65%, respectively) (Fig. 2C). Taken together, these data suggest that TcfC is broadly distributed across serovars in most subspecies *enterica* clades, with plasmid-mediated acquisition occurring in some cases, especially for divergent copies of TcfC or for the acquisition of other α-fimbriae from other genera.

### The β-fimbrial usher StjB is detected in representative isolates of all subspecies *enterica* phylogenetic clades except for clade C and section Typhi.

**FIG 3:**
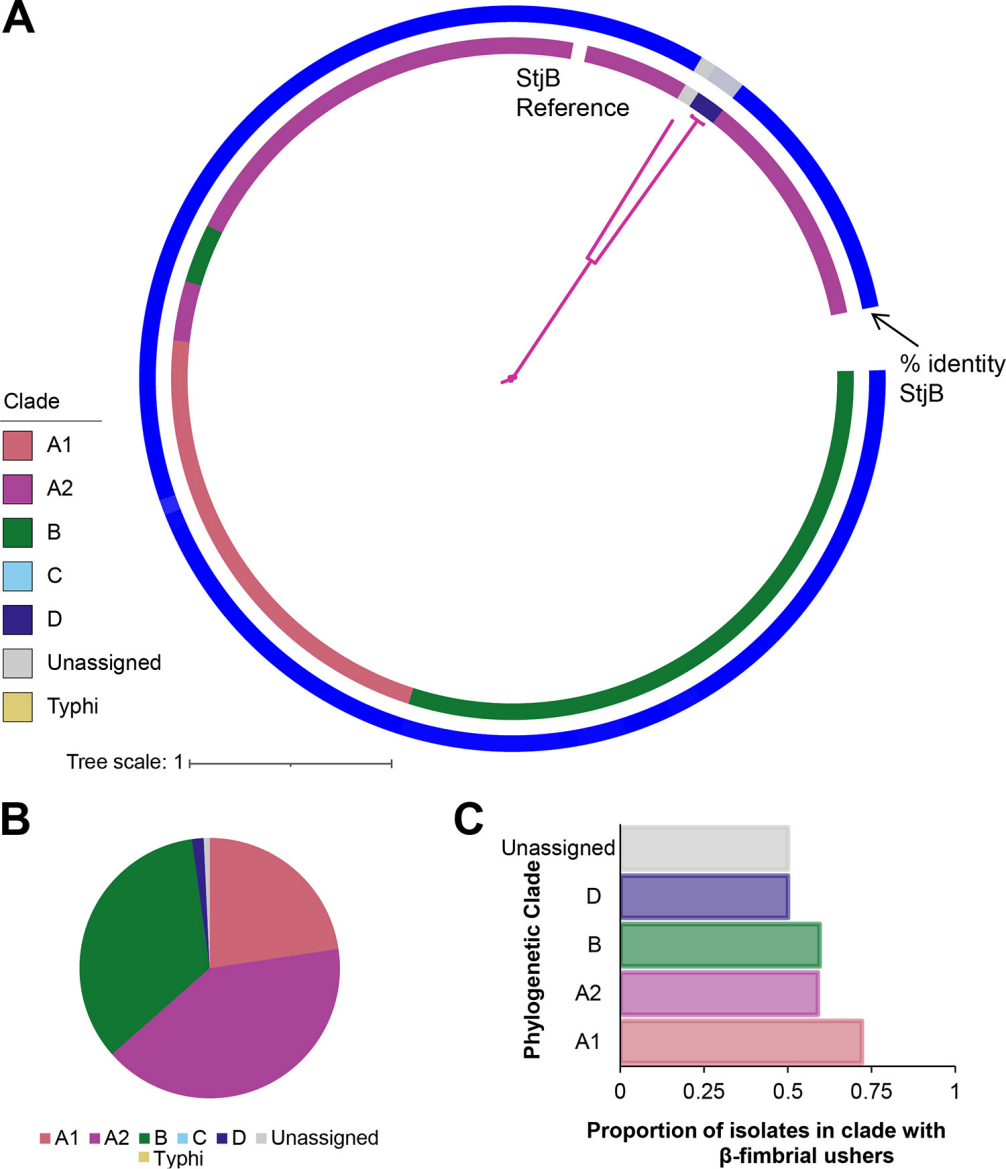
Distribution of the β-fimbrial usher StjB. (A) Phylogeny inferred from amino acid sequences from 137 β-fimbrial ushers and StjB from *S.* Typhimurium LT2 (used as a reference). Bootstrap values represent the percentage of times that the clustering observed in the phylogenetic tree was reproduced among the 1,000 UF bootstrap repetitions. The innermost colored strip represents the phylogenetic clade of the isolate, while the outermost strip represents the percent amino acid identity between the usher and the StjB reference sequence (range, 74 to 100%). Phylogeny was inferred using the WAG+F+R2 model (selected based on the BIC score). (B) Proportion of fimbriae from isolates in each phylogenetic clade. “Unassigned” refers to Salmonella serovars Poano and Lattenkamp, which could not be assigned to a major phylogenetic clade. (C) Proportion of isolates in each phylogenetic clade in which StjB was detected. StjB was not detected in any isolates in clade C or section Typhi, and therefore, these bars are omitted from the chart.

The Stj fimbria, first identified in *S.* Typhimurium strain LT2 (33), was the only known β-fimbria encoded by subspecies *enterica* (18, 26). In agreement, among the 137 β-fimbrial ushers identified here in the subspecies *enterica* isolates, all sequences shared >70% amino acid identity with the StjB reference (Fig. 3A), with most sharing 99 to 100% identity with the StjB reference, suggesting that StjB is the only β-fimbrial usher encoded by subspecies *enterica* serovars (all β-fimbrial ushers were classified as StjB here [including divergent sequences sharing lower % ID with the reference StjB] to avoid introducing additional fimbriae). The most divergent StjB included sequences from two isolates representing clade D serovars (*S*. Brazzaville and *S*. Westminster) and an isolate representing *S.* Lattenkamp, which could not be assigned to a phylogenetic clade. StjB was not detected in any of the clade C (i.e., 0/5 serovars) or section Typhi (i.e., 0/14 serovars) isolates (Fig. 3B and C), suggesting that this usher has been lost in these clades. Clade A1 had the highest proportion of isolates with StjB (72%), while StjB was detected in approximately 50 to 60% of isolates in clades A2, B, and D. None of the StjB sequences compared here were detected on contigs associated with plasmids, suggesting that this fimbria is chromosomally encoded. Together, these results suggest that StjB remains the only β-fimbria encoded by subspecies *enterica* serovars.

### Ushers belonging to the γ-fimbrial family account for 70% of all CU fimbrial ushers detected.

**FIG 4:**
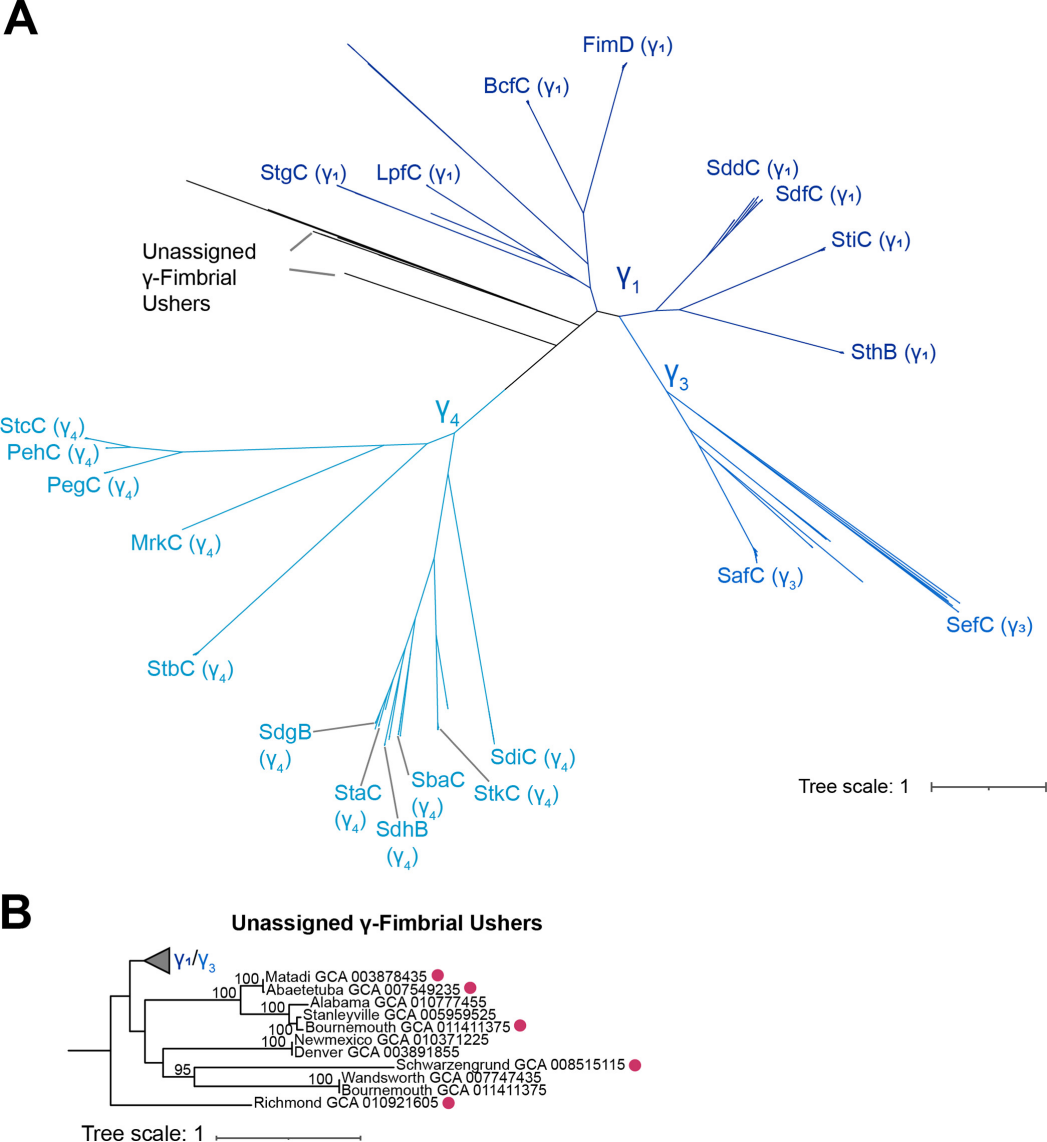
Distribution of the γ-fimbrial ushers. (A) Unrooted phylogeny for 2,031 γ-fimbrial ushers and 21 reference ushers (shown as labeled branches in the phylogeny) (see Table 2 for information regarding the source of the reference sequence). Clades (γ_1_ to γ_4_) were assigned as proposed previously (18); reference sequences for γ_2_-fimbriae were not included, as these fimbriae have been found only in non-Salmonella isolates (18, 26). A total of 10 ushers assigned to the γ-fimbrial family could not be assigned to a clade based on sequence homology with known ushers; branches representing these divergent γ-fimbrial ushers are shown in black. Bootstrap values represent the percentage of times that the clustering observed in the phylogenetic tree was reproduced among the 1,000 UF bootstrap repetitions. Phylogeny was inferred using the JTT+F+R7 model (selected based on the BIC score). (B) Pruned phylogeny showing the phylogenetic relationship of the 11 divergent fimbrial ushers that could not be confidently assigned to a clade. Magenta circles shown external to the labeled branches represent usher sequences that were associated with carriage on a plasmid. Bootstrap values of ≥95 are shown.

Most of the previously reported CU fimbriae in subspecies *enterica* belong to the γ-fimbrial family (18, 25, 26), including the most widely studied CU fimbria, the type I fimbria Fim (25, 34). Altogether, we detected 2,031 CU fimbrial ushers that clustered with γ-fimbriae reference sequences (Fig. 4A). Given the number of fimbriae in this family, Nuccio and Bäumler previously proposed that the family be subclassified into groups γ_1_ through γ_4_ (18).

All but 11 fimbrial ushers clustered with reference sequences and could be further classified into subfamilies γ_1_, γ_3_, and γ_4_; these 11 fimbrial ushers represented divergent ushers that shared low sequence identity (53 to 71% sequence identity) with all γ-fimbria reference sequences, including 5 sequences that mapped to contigs belonging to plasmids and 2 sequences from isolates representing clade C Salmonella serovars Denver and Newmexico (Fig. 4B). Translated nucleotide BLAST (tBLASTn) searches of these ushers suggested that some are found in other Gram-negative genera, including Citrobacter portucalensis and Escherichia coli, while others shared high identity and coverage scores with other Salmonella plasmid sequences in the database (Data Set S1); all ushers mapped to fimbrial gene clusters that included additional coding sequences with annotations such as “fimbrial-type adhesion domain superfamily” and “pilus assembly chaperone” (see Data Set S1 for annotations of additional coding sequences detected in the proximity of these divergent fimbrial ushers).

**FIG 5:**
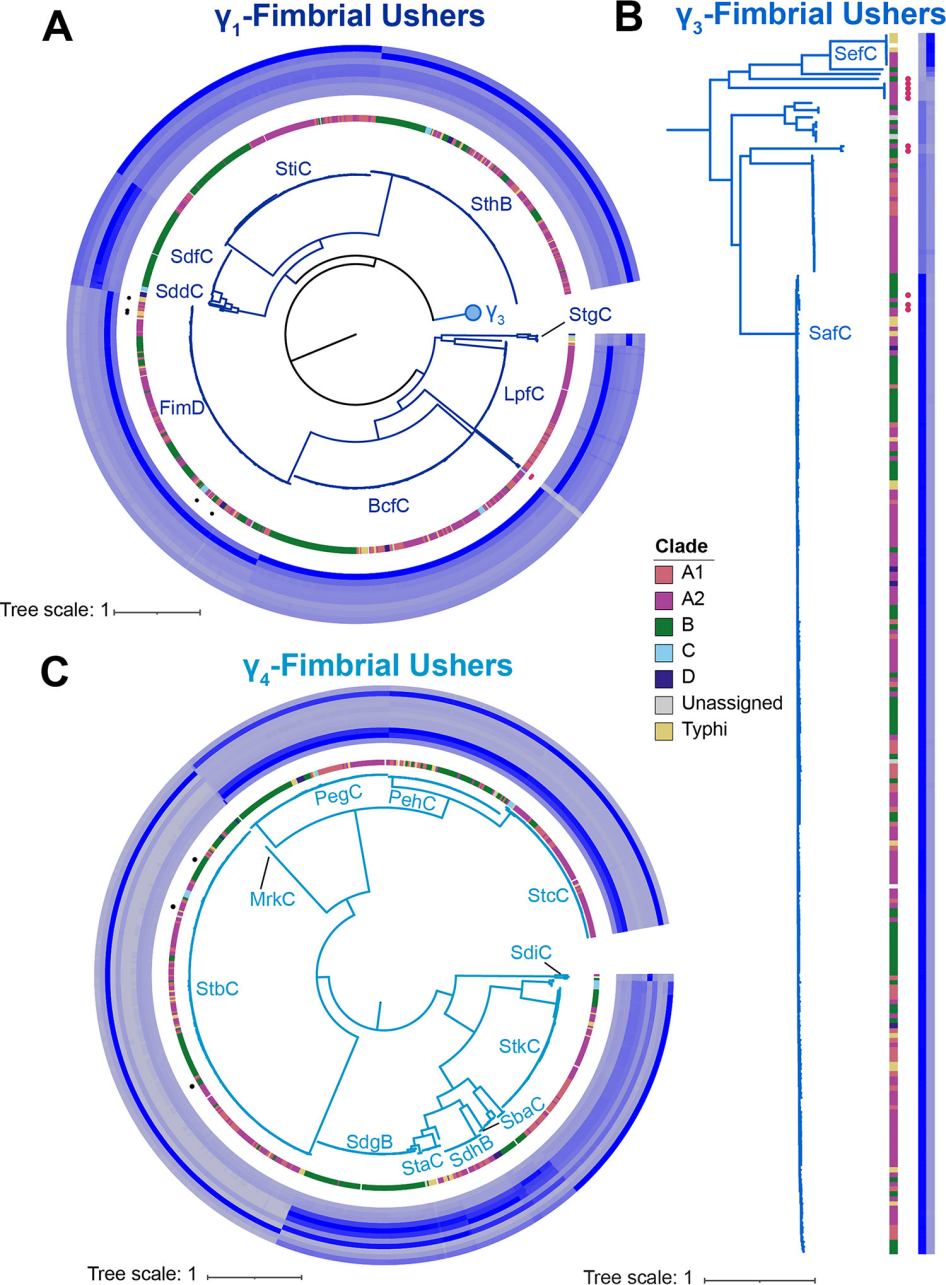
Identity of γ_1_-, γ_3_-, and γ_4_-fimbrial ushers. All phylogenies are inferred from amino acid sequences; bootstrap values represent the percentage of times that the clustering observed in the phylogenetic tree was reproduced among the 1,000 UF bootstrap repetitions. The innermost colored strip shows the phylogenetic clade of the isolate from which the usher sequence was extracted. Filled-in circles represent usher sequences that were associated with carriage on a plasmid (pink) or prophage (black). “Unassigned” refers to isolates representing Salmonella serovars Poano and Lattenkamp, which could not be assigned to a major phylogenetic clade. Phylogenies were inferred using the JTT+F+R7 model (selected based on the BIC score). (A) Phylogenetic tree of γ_1_-fimbrial ushers. Colored strips represent percent identities for reference sequences with (inner to outer circles) BcfC, FimD, LpfC, SddC, SdfC, StgC, SthB, and StiC (see Table 2 for information regarding the reference sequences used). (B) Phylogenetic tree of γ_3_-fimbrial ushers, with strips representing percent identities to SafC (inner strip) and SefC (outer strip) reference sequences. (C) Phylogenetic tree of γ_4_-fimbrial ushers. Colored strips represent percent identities for MrkC, PegC, PehC, SbaC, SdgB, SdhB, SdiC, StaC, StbC, StcC, and StkC reference sequences (inner to outer circles). Percent identity ranged from 50 to 100%.

The γ_1_ subfamily included the largest number of ushers (1,102 ushers) (Fig. 5A). Ushers BcfC, FimD, and SthB were detected in ≥86% of isolates in each phylogenetic clade, while the distributions of LpfC, SddC/SdfC (i.e., ushers shared similar %IDs with both SddC and SdfC and therefore were classified as SddC/SdfC), and StiC were more clade specific. For example, LpfC was found only in isolates representing serovars in clades A1 and A2 and in the isolate representing section Typhi serovar Stanleyville, while SddC/SdfC was detected only in isolates from clades B, C, and D. StgC is present in some isolates in clades C and D and section Typhi. Finally, 4 of the sequences within the γ_1_ subfamily represented divergent sequences with low sequence homology to all γ_1_ subfamily reference sequences (60 to 65% amino acid identity); for isolates representing Salmonella serovars Okatie (clade A2) and Sanjuan (clade C) these ushers were associated with carriage on a plasmid, while for isolates representing Salmonella serovars Aqua (clade A2) and Weslaco (clade C), the association of these fimbrial ushers with carriage on a plasmid could not be confirmed (Fig. 5A). FimD was the only usher detected within sequences assigned to a prophage, although in all cases but one (isolate representing section Typhi serovar Lexington), these prophages were not intact (Table 1), suggesting that the acquisition of this fimbria may have originally been mediated by a prophage.

**TABLE 1:** Description of usher proteins associated with carriage on a prophage

Clade	Genome	Usher^*a*^	Length^*b*^	Prophage^*c*^	Prophage genome completeness^*d*^
A1	Worthington_GCA_008145485	FimD	Full	PHAGE_Entero_UAB_Phi20_NC_031019	Incomplete
A2	Kibusi_GCA_004225845	StbC	Full	PHAGE_Entero_UAB_Phi20_NC_031019	Incomplete
A2	Kintambo_GCA_008413925	FimD	Partial	PHAGE_Salmon_epsilon34_NC_011976	Incomplete
A2	Kottbus_GCA_003878215	FimD	Partial	PHAGE_Salmon_epsilon34_NC_011976	Incomplete
A2	Ouakam_GCA_010700005	FimD	Partial	PHAGE_Salmon_epsilon34_NC_011976	Incomplete
B	Beaudesert_GCA_004192575	SafC	Partial	PHAGE_Stx2_c_Stx2a_F451_NC_049924	Questionable
B	Kisarawe_GCA_010794395	SafC	Partial	PHAGE_Stx2_c_1717_NC_011357	Questionable
B	Montevideo_GCA_008824545	FimD	Partial	PHAGE_Salmon_118970_sal4_NC_030919	Questionable
B	Oranienburg_GCA_005566805	FimD	Partial	PHAGE_Photob_PDCC_1_NC_048821	Questionable
B	Pomona_GCA_010511755	StbC	Full	PHAGE_Salmon_vB_SosS_Oslo_NC_018279	Questionable
B	Rubislaw_GCA_006637005	StbC	Full	PHAGE_Entero_P4_NC_001609	Incomplete
C	Newmexico_GCA_010371225	FimD	Full	PHAGE_Stx2_c_1717_NC_011357	Questionable
Typhi	Cerro_GCA_007747355	FimD	Full	PHAGE_Salmon_epsilon34_NC_011976	Incomplete
Typhi	Kiambu_GCA_016027635	FimD	Partial	PHAGE_Salmon_118970_sal4_NC_030919	Incomplete
Typhi	Lexington_GCA_006519135	FimD	Full	PHAGE_Salmon_Fels_1_NC_010391	Intact
Typhi	Paratyphi_A_GCA_005604015	FimD	Full	PHAGE_Salmon_epsilon34_NC_011976	Incomplete
S. bongori	bongori_GCA_013138145	Κ_Other	Full	PHAGE_Entero_HK633_NC_019719	Incomplete
IIIa	IIIa_GCA_008431985	FimD	Partial	PHAGE_Entero_mEp237_NC_019704	Questionable
IIIa	IIIa_GCA_004222315	FimD	Partial	PHAGE_Ralsto_RSF1_NC_028899	Questionable
IV	IV_GCA_010940595	StdB	Full	PHAGE_Phage_Gifsy_1_NC_010392	Questionable
IV	IV_GCA_008524605	StdB	Full	PHAGE_Phage_Gifsy_1_NC_010392	Questionable
VII	VII_GCA_010164295	FimD	Full	PHAGE_Escher_500465_2_NC_049343	Incomplete

a Identity assigned based on alignment scores with reference usher proteins and/or phylogenetic analysis of aligned sequences of unknown usher proteins with a reference sequence.

b Length of the usher extracted from Prokka-annotated sequences of prophage regions identified by Phaster.

c Prophage identity assigned by Phaster.

d Genome completeness score assigned by Phaster. Intact prophage genomes are assigned a score of >90, while questionable and incomplete represent prophage genomes having scores of 70 to 90 and <70, respectively. Scores are assigned based on the number of coding sequences in the region that match a known phage, the number of predicted proteins in the region, the size of the region, and the presence of phage-related keywords.

The γ_3_ subfamily was the smallest within the γ-fimbriae and contained the SafC and SefC reference sequences and 252 additional ushers from isolates representing subspecies *enterica* serovars (Fig. 5B). Most of the CU fimbrial ushers (*n* = 203) in this subfamily shared ≥98% amino acid identity with the SafC reference (Fig. 5B); this usher was detected in isolates in every major phylogenetic clade except for those representing clade C serovars, suggesting that SafC is relatively conserved in subspecies *enterica*. Only 6 of the ushers in this subfamily shared high (≥97%) amino acid identity with the SefC reference; one usher from an isolate representing clade B serovar Pomona encoded a more divergent copy (80% identity with the SefC reference). The remaining 42 ushers shared lower percent amino acid identity scores with the SafC and SefC references (i.e., referred to as SafC/SefC), including 7 ushers that were associated with plasmid sequences (Fig. 5B).

Finally, one-third of the CU fimbrial ushers (*n* = 666) were assigned to the γ_4_ subfamily (Fig. 5C). StbC and PegC were found in multiple isolates representing all major phylogenetic clades. The remaining ushers showed clade-specific distributions or were variably present in some clades. Examples of ushers with a clade-specific distribution include (i) SbaC and SdgB, which are associated almost exclusively with clade B serovars, and (ii) StaC, which is present in 57% of isolates representing serovars in section Typhi and just 4 isolates representing serovars in clade A2. Ushers PehC, StcC, and StkC were present in at least some isolates in most phylogenetic clades. We did not find any isolates that had the MrkC fimbrial usher (26), and the SdiC usher was detected only in isolates representing Salmonella serovars Infantis (clade A2) and Florida (clade B), suggesting that these fimbrial ushers are rare among subspecies *enterica* isolates. We found 3 full-length StbC sequences that mapped to prophage regions in isolates representing Salmonella serovars Kibusi (clade A2), Pomona (clade B), and Rubislaw (clade B); however, similar to FimD, the distribution of this fimbria throughout the majority of the isolates suggests that prophage-mediated acquisition of StbC may have occurred, such as in a common ancestor of S. enterica subsp. *diarizonae*, which was proposed previously to be the predicted acquisition for this fimbria (30).

Overall, among the 2,031 γ-fimbrial ushers examined here, few represent potentially novel fimbrial ushers, although ushers associated with carriage on a plasmid or those associated with phylogenetic clades C and D represent divergent sequences.

### Fae κ-fimbrial ushers are broadly distributed among representative isolates in all major subspecies *enterica* phylogenetic clades except for clade D, while PefC is rare and always associated with carriage on a plasmid.

**FIG 6:**
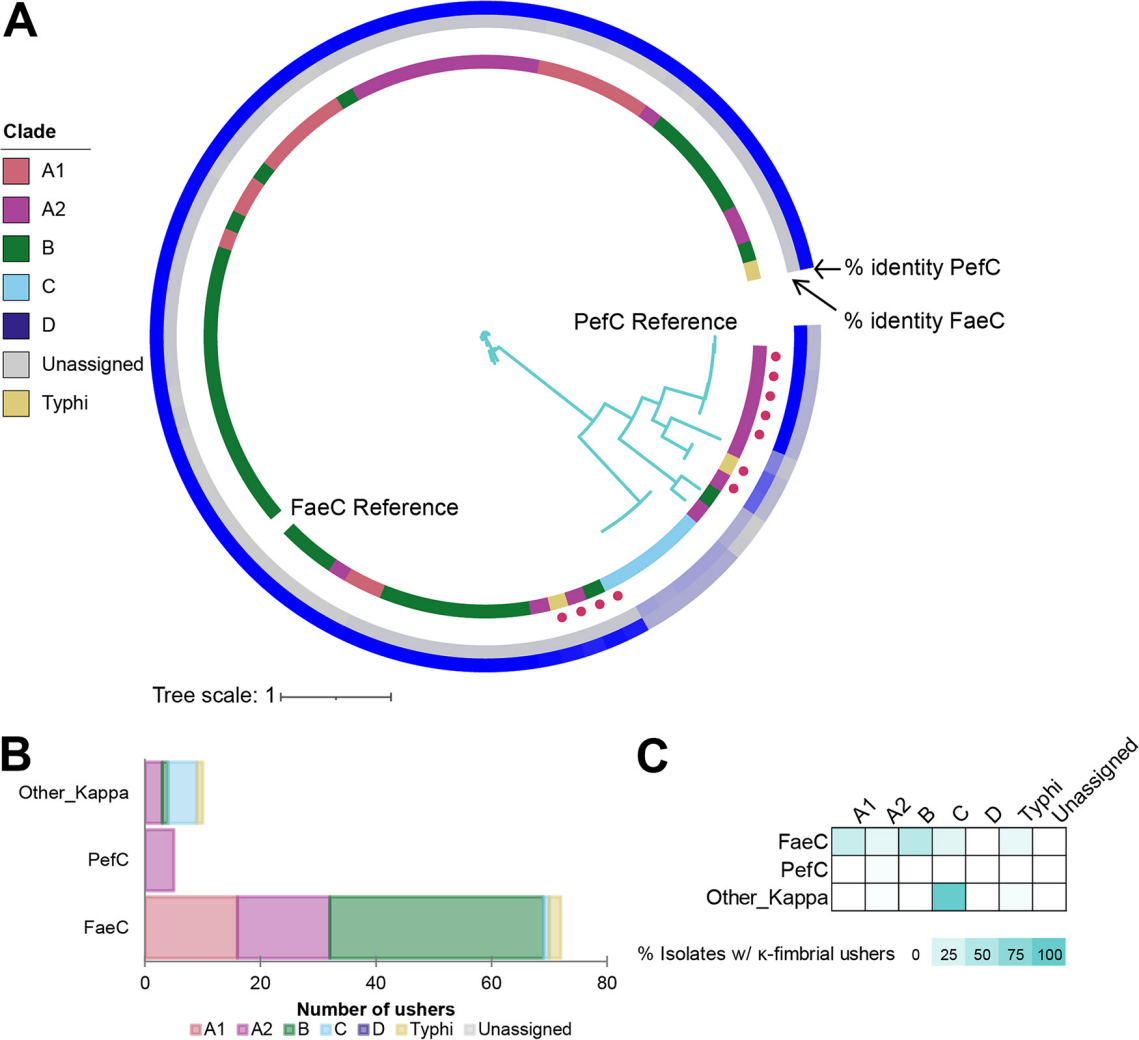
Distribution of the κ-fimbrial ushers. (A) Phylogeny inferred from amino acid residues from 87 κ-fimbrial ushers with the PefC (*S.* Typhimurium strain LT2) and FaeC (*S.* Poona strain NCTC4840) references. Bootstrap values represent the percentage of times that the clustering observed in the phylogenetic tree was reproduced among the 1,000 UF bootstrap repetitions. Colored strips shown external to the tree represent the percent identities to the FaeC (inner circle) (range, 63 to 100%) and PefC (outer circle) (range, 67 to 100%) references; blue represents fimbrial sequences with higher percent identity, while fimbriae with a gray strip share a lower percent identity with reference sequences. Magenta circles represent ushers that were detected on contigs associated with plasmids. “Unassigned” refers to isolates representing Salmonella serovars Poano and Lattenkamp, which could not be assigned to a major phylogenetic clade. Phylogeny was inferred using the LG+F+G4 model (selected based on the BIC score). (B) Distribution of κ-fimbriae from isolates in each phylogenetic clade. (C) Proportion of isolates in each phylogenetic clade having at least one κ-fimbria detected. Other_Kappa represents divergent copies of fimbriae that could not be assigned an identity.

Two plasmid-encoded κ-fimbriae had been previously described in subspecies *enterica* serovars: (i) the plasmid-encoded fimbria (Pef) carried on the Salmonella virulence plasmid (35–37) and (ii) the Fae (also known as K88) fimbria, which is also encoded by some E. coli strains (38). Among the 87 CU fimbrial usher proteins assigned to the κ-fimbria family, 72 shared high amino acid identity with FaeC (≥95% identity with the FaeC reference), compared to just 5 that shared high identity (100%) with the PefC reference (Fig. 6A). The remaining 10 sequences represented divergent sequences of PefC or FaeC or a potentially novel κ-fimbrial usher, including (i) 5 sequences from clade C isolates that shared 100% identity with other clade C isolates but just 69% and 73 to 74% identities with the FaeC and PefC references, respectively, and (ii) 5 sequences from isolates in multiple clades having 72 to 85% identity with PefC and 63 to 67% identity with the FaeC references. All 5 fimbrial ushers that shared 100% identity with the PefC reference were identified in clade A2 serovars, while isolates having FaeC were more evenly distributed across clades A1, A2 and B and section Typhi (Fig. 6B and C).

All 5 PefC sequences were assigned to contigs associated with plasmids (Fig. 6A), presumably representing the Salmonella virulence plasmid (as suggested by the presence of other known virulence factors [SpvB and SpvC] associated with this plasmid [36]). In contrast, just 4 of the FaeC sequences were associated with contigs assigned to plasmids (Fig. 6A). While this may suggest that FaeC is chromosomally encoded, it is important to note that plasmids sequenced by short-read sequencing are inherently challenging to assemble. Of the divergent κ-fimbrial ushers that may represent ancestral sequences of PefC or FaeC, 2 were associated with plasmid contigs in isolates representing *S.* Inverness (clade A2) and *S.* Mississippi (section Typhi) (Fig. 6A).

Together, our results suggest that FaeC is broadly distributed among subspecies *enterica* isolates, with at least one isolate from all major phylogenetic clades except for clade D carrying FaeC. Conversely, PefC is associated with only 5 serovars, all of which are in clade A2 and are always associated with a plasmid.

### The StdB π-fimbrial usher is widely distributed across isolates in all clades, while the SbbB, StfC, and SteB fimbrial ushers are associated with specific phylogenetic clades.

**FIG 7:**
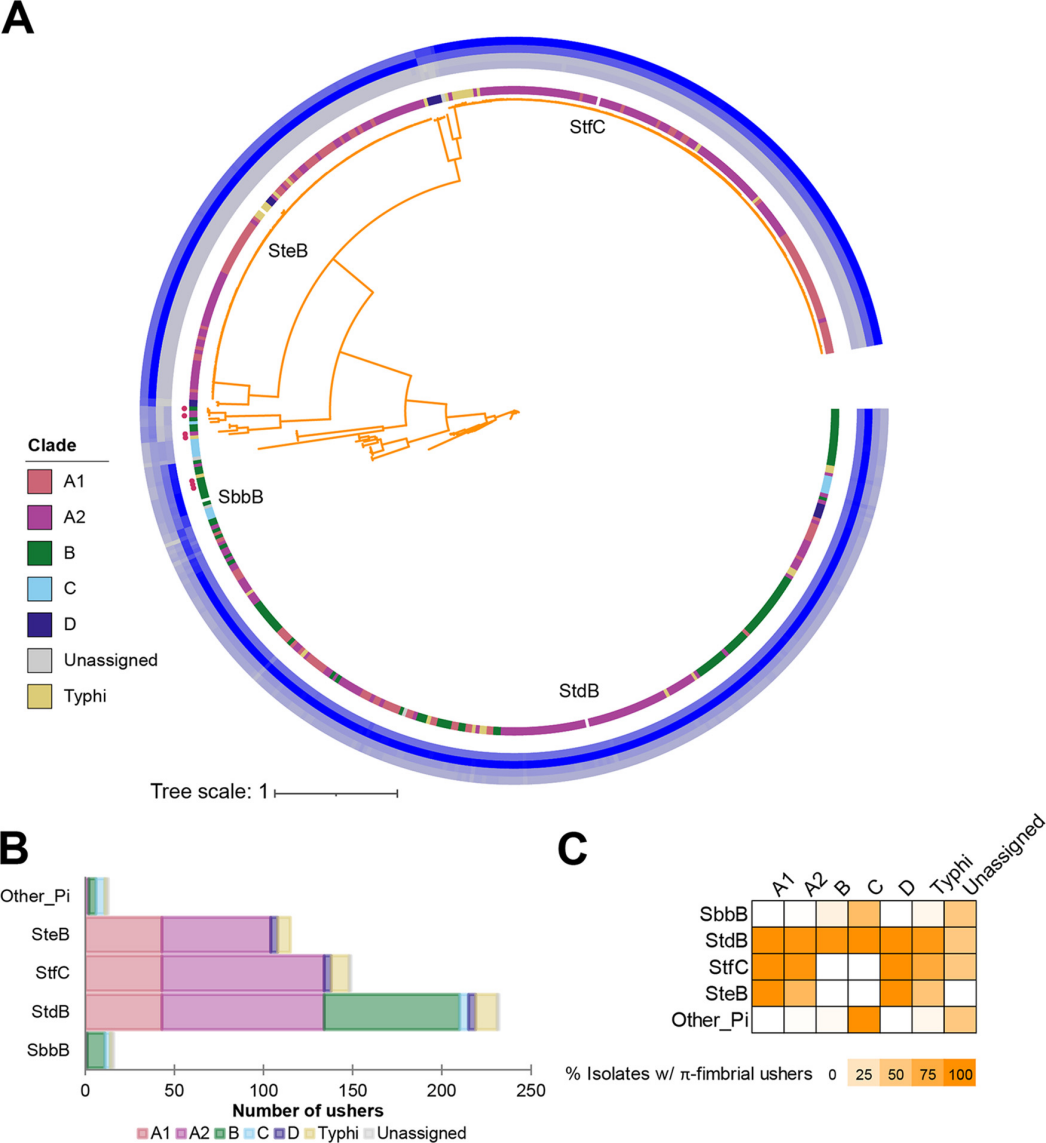
Distribution of the π-fimbrial ushers. (A) Phylogeny inferred from amino acid sequences for 536 π-fimbria and 4 reference sequences (SbbC [S. bongori strain NCTC12419], StdB and StfC [*S.* Typhimurium strain LT2], and SteB [*S.* Typhi strain CT18]). Bootstrap values represent the percentage of times that the clustering observed in the phylogenetic tree was reproduced among the 1,000 UF bootstrap repetitions. Colored strips shown external to the tree represent the percent identities to the SbbC (inner circle), StdB (second inner circle), SteB (third inner circle), and StfC (outer circle) references (range, 60 to 100%); blue shows isolates with higher percent identity, while isolates with a gray strip have lower percent identity. Magenta circles represent usher sequences detected on plasmid contigs. “Unassigned” refers to isolates representing Salmonella serovars Poano and Lattenkamp, which could not be assigned to a major phylogenetic clade using the techniques described here. Phylogeny was inferred using the LG+F+R5 model (selected based on the BIC score). (B) Number of π-fimbrial ushers from isolates in each phylogenetic clade. (C) Heat map representation of the percentage of isolates in each phylogenetic clade encoding each π-fimbrial usher. Other_Pi represents π-fimbrial ushers that could not be assigned to a known π-fimbrial reference sequence and therefore represent divergent π-fimbrial ushers.

At least 6 different π-fimbriae had been reported previously (26): Sbb in S. bongori (26, 31), Sdl and Sdk in subspecies *diarizonae* (26), Std and Ste in *S.* Typhi (27), and Stf in *S.* Typhimurium (33). We found that SbbB is also found in at least one serovar in all clades except for clades A1 and D, suggesting that this fimbria is not specific to S. bongori, although it is rare among the subspecies *enterica* isolates examined here (present in 16 out of 242 isolates) (Fig. 7A). StdB was the most widely distributed π-fimbrial usher and was detected in 232 out of 242 isolates, with at least one isolate from each phylogenetic clade encoding StdB (Fig. 7A to C). StfC and SteB were not detected in any of the isolates in clades B and C or the 2 isolates representing serovars that could not be confidently assigned to a clade despite being detected in the majority of serovars in clades A1, A2, and D and section Typhi. Finally, we detected 15 π-fimbrial ushers that shared lower amino acid identities with the 4 reference sequences (66 to 75%), suggesting that these sequences represent divergent copies of previously identified fimbriae or perhaps novel fimbriae acquired from other genera. Although the majority of these were found in isolates in phylogenetic clade C, some were associated with carriage on plasmids for serovars in other phylogenetic clades (isolates representing Salmonella serovars Glostrup, Kottbus, Lexington, and Richmond), which formed a subclade that shared an ancestor with the SteB and StfC reference sequences.

Together, our results suggest that the StdB π-fimbria is conserved across most phylogenetic clades of subspecies *enterica*, while Stf and Ste fimbriae show a clade-specific distribution, as these fimbriae were not detected in any of the clade B or C serovars. Finally, SbbB, although rarer than other π-fimbriae, is found in at least some serovars in most clades, with at least some sequences being associated with carriage on a plasmid.

### The majority of fimbrial ushers are found in multiple non-*enterica* subspecies serovars, suggesting that they were most likely acquired prior to the divergence of subspecies *enterica*.

**FIG 8:**
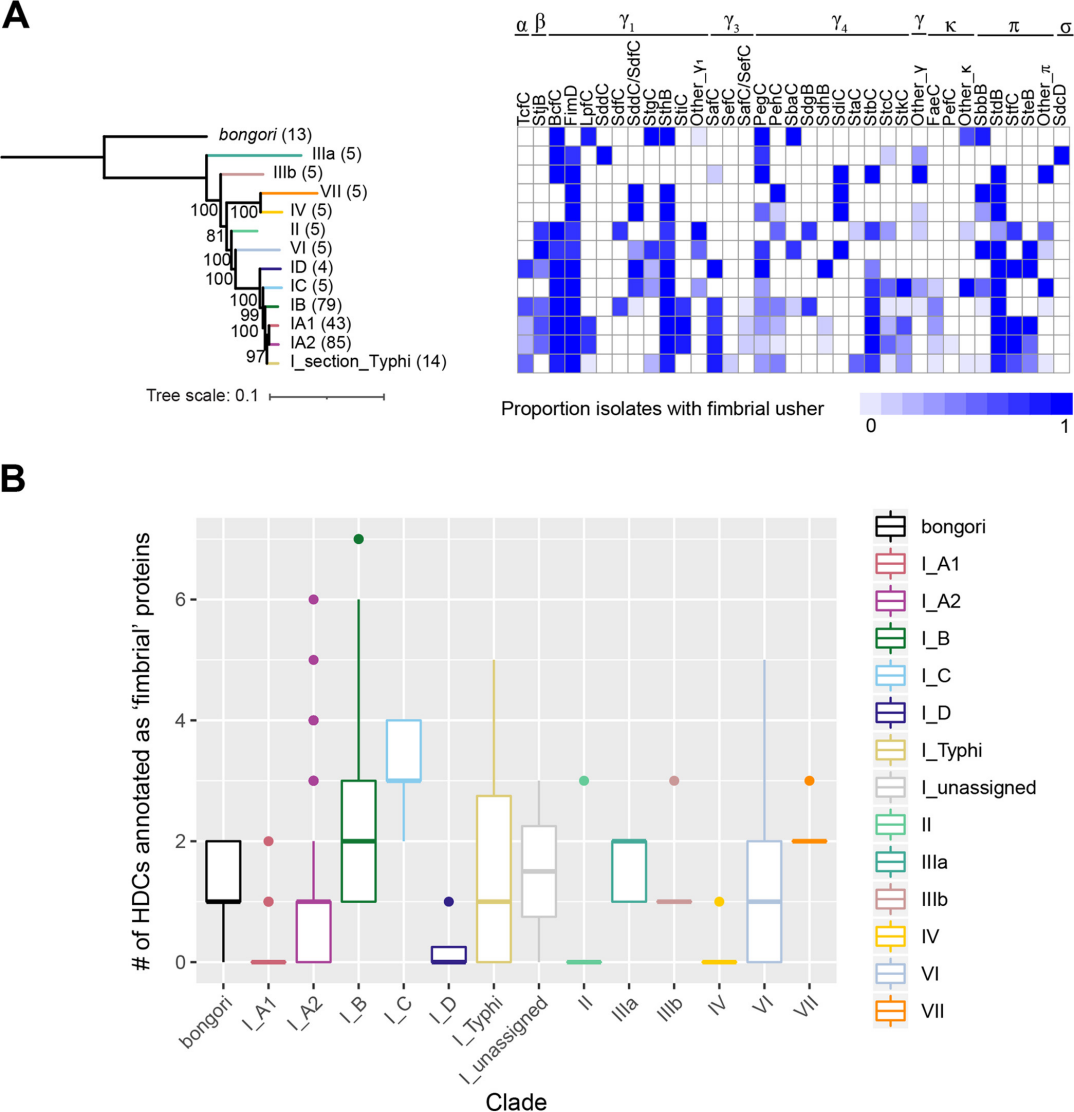
Comparison of CU fimbrial ushers in Salmonella species isolates and major S. enterica subsp. *enterica* phylogenetic clades. (A) Core SNP phylogeny was inferred for representative isolates of each major phylogenetic group. Isolates were selected based on their assemblies having the lowest number of contigs among isolates in their respective phylogenetic groups. Bootstrap support values listed represent the averages from 1,000 repetitions; only support values of >80 are shown. Phylogeny was inferred using isolates representing each phylogenetic clade (S. bongori GCA_003522735.1, IIIa GCA_016029015.1, IIIb GCA_005803545.1, VII GCA_018338515.1, IV GCA_010940595.1, II GCA_019542375.1, VI GCA_018032985.1, ID Montaigu GCA_009247085.1, IC Newmexico GCA_010371225.1, IB Horsham GCA_010441095.1, IA2 Ealing GCA_010176655.1, IA1 Blockley GCA_010175875.1, and IA section Typhi Kiambu GCA_016027635.1). Numbers to the right of the leaf label show the number of isolates in the phylogenetic group (i.e., number of isolates upon which the data in the heat map are based). The heat map summarizes the proportion of isolates in the phylogenetic group that encode a given usher (as determined by sequence identity with reference sequences and phylogenetic clustering [see Data Set S3 in the supplemental material]). For SafC and SefC, divergent sequences (i.e., those that shared lower sequence identity with reference sequences) are designated “SafC/SefC,” and divergent sequences of SddC and SdfC are similarly designated “SddC/SdfC.” (B) Comparison of the numbers of hypothetically disrupted coding sequences (HDCs) annotated as “fimbrial” among all Salmonella species isolates. HDCs were identified in assemblies annotated with PGAP; as the presence of a premature stop codon may or may not represent proteins with a loss of function, the term HDC is used (39). “Fimbrial” includes all coding sequences in a fimbrial gene cluster (i.e., not just usher proteins). The presence of a fimbrial HDC may or may not affect the function of the fimbria.

To better understand the evolutionary events shaping the acquisition/loss of CU fimbrial ushers in the subspecies *enterica* isolates examined here, we next characterized the CU fimbrial ushers of representative isolates for serovars of S. bongori and other non-*enterica* subspecies (Fig. S1A). Similar to what was observed for subspecies *enterica*, members of the γ_1_- and γ_4_-fimbrial families were also the most abundant ushers detected in the non-*enterica* subspecies isolates. Among γ_1_ ushers, BcfC, FimD, and SthB were common throughout isolates in the non-*enterica* subspecies clades, although clade-specific losses most likely occurred in select subspecies, such as (i) the loss of BcfC in S. enterica subsp. *houtenae* and VII isolates and (ii) the loss of SthB in S. enterica subsp. *arizonae* and *diarizonae* isolates (Fig. 8A and Fig. S1B). In contrast to what was observed in subspecies *enterica* isolates, SafC was detected in only a single subspecies *diarizonae* isolate (Fig. S1B) and was not associated with carriage on a prophage or plasmid, and SefC was not detected in any of the non-*enterica* subspecies isolates, suggesting that γ_3_ ushers were likely acquired with the divergence of subspecies *enterica* (Fig. 8A). Finally, the γ_4_ ushers PegC, PehC, and SdiC were broadly distributed among the non-*enterica* subspecies isolates, supporting their acquisition by either S. bongori (PegC) or another S. enterica subspecies (SdiC by IIIb or PehC by VII) (Fig. 8A); the remaining γ_4_ ushers were present in fewer non-*enterica* subspecies clades, suggesting either their acquisition and subsequent loss by most non-*enterica* subspecies clades or their acquisition by select subspecies.

10.1128/msystems.00115-22.1 FIG S1 Identity of fimbrial ushers in non-*enterica* subspecies isolates. (A) Families of 358 CU fimbrial ushers identified in non-*enterica* subspecies isolates representing unique serovars of S. bongori (13 isolates), and S. enterica subspecies (II to VII) (5 isolates each). Some ushers in the γ-fimbrial family could not be further assigned to a subfamily (labeled “unassigned” in the figure) due to low amino acid similarity with reference sequences. Clades are shaded to represent the CU fimbrial family to which the usher proteins belong. (B) Phylogeny of amino acid sequences for 252 γ-fimbrial ushers in non-*enterica* subspecies isolates. Leaves representing fimbrial usher reference sequences are labeled. The innermost ring represents the clade of the isolate. Branches are colored based on the isolate belonging to the γ_1_ (dark blue), γ_3_ (medium blue), or γ_4_ (light blue) subfamily; note that reference sequences for γ_2_-fimbrial ushers were not included, as these ushers had not been previously detected in Salmonella spp. Phylogeny was inferred using the LG+F+R6 model (selected based on the BIC score). Colored strips shown external to the clade represent the shared percent identity of a fimbrial usher with a given reference (shown from innermost to outermost [BcfC, FimD, LpfC, SddC, SdfC, StgC, SthB, StiC, SafC, SefC, MrkC, PegC, PehC, SbaC, SdgB, SdhB, SdiC, StaC, StbC, StcC, and StkC]). Download FIG S1, TIF file, 2.8 MB. Copyright © 2022 Cheng et al. 2022 Cheng et al. https://creativecommons.org/licenses/by/4.0/ This content is distributed under the terms of the Creative Commons Attribution 4.0 International license.

10.1128/msystems.00115-22.8 DATA SET S3 Identifications and fimbrial families of all CU fimbrial ushers characterized in this study. Download Data Set S3, XLSX file, 0.1 MB. Copyright © 2022 Cheng et al. 2022 Cheng et al. https://creativecommons.org/licenses/by/4.0/ This content is distributed under the terms of the Creative Commons Attribution 4.0 International license.

β-Fimbrial ushers were detected only in S. enterica subsp. *salamae* and *indica* genomes (Fig. S2A), with most sharing low sequence identity with the StjB reference usher (range, 62 to 75% identity); despite having low sequence identity, we elected to classify these divergent fimbriae as StjB to avoid introducing novel fimbriae here, although future analyses examining their evolutionary relationship to StjB will be necessary. Ushers belonging to the κ-fimbrial family were found exclusively in S. bongori and one subspecies *salamae* isolate, suggesting that members of this fimbrial family are not common in non-*enterica* subspecies clades (Fig. S2B). For S. bongori and the subspecies *salamae* isolate encoding κ-fimbrial ushers, follow-up analyses did not locate these ushers on plasmid sequences, suggesting that these ushers may be chromosomally encoded in non-*enterica* subspecies isolates. In general, the κ-fimbrial ushers shared a slightly higher percent identity with PefC than with FaeC references (73% versus 68% identity, respectively) (Fig. S2B). Among the π-fimbrial ushers, StdB was the most common, being detected in isolates of all subspecies except for subspecies *arizonae* and S. bongori, while SteB and StfC were detected only in isolates of subspecies *indica* and *salamae*, respectively (Fig. S2C). Some of the π-fimbrial ushers detected in subspecies *salamae* and *diarizonae* isolates shared low sequence identity with reference sequences (Fig. S2C). The σ-fimbrial usher SdcD was unique to isolates of subspecies *arizonae* (Fig. S2D), corroborating the results of previous studies (26).

10.1128/msystems.00115-22.2 FIG S2 Identities of β-, κ-, π-, and σ-fimbrial ushers in non-*enterica* subspecies isolates. Shown are phylogenies of amino acid sequences of fimbrial ushers assigned to β-fimbrial (A), κ-fimbrial (B), π-fimbrial (C), and σ-fimbrial (D) ushers. The innermost colored strips represent the clade of the isolate from which the usher sequence was extracted (see the key at the bottom right). Strips to the right of the clade designation show the shared percent identity of the usher with the reference sequence (darker blue colors represent higher percent identity with the reference, while sequences shown in gray represent those with lower percent identity). For each phylogeny, the substitution model used was based on the model having the lowest BIC score; bootstrap support values represent the averages from 1,000 repetitions. In panel C, the percent identity scores are shown for SbbB (innermost ring), StdB, SteB, and StfC (outermost ring). Labeled branches represent the reference usher sequences. Download FIG S2, TIF file, 1.3 MB. Copyright © 2022 Cheng et al. 2022 Cheng et al. https://creativecommons.org/licenses/by/4.0/ This content is distributed under the terms of the Creative Commons Attribution 4.0 International license.

Few of the ushers identified in non-*enterica* subspecies isolates mapped to mobile elements (Fig. S3), suggesting that the majority of these fimbriae are chromosomally encoded. None of the β-, γ_3_-, or γ_4_-fimbriae were detected on prophage or plasmid regions, while only one of the κ-fimbrial ushers (in a single S. bongori isolate) (Fig. S3) mapped to a prophage region, and 4 of the FimD sequences mapped to prophage regions in subspecies *arizonae* and VII isolates, suggesting that the acquisition of this fimbria may have been mediated by a prophage (Table 1). Ushers detected on plasmid regions included (i) 3 γ-fimbrial ushers with low sequence identity to known γ_1_, γ_3_, and γ_4_ sequences and (ii) a single StdB fimbrial usher encoded by a subspecies *diarizonae* isolate (Fig. S3).

10.1128/msystems.00115-22.3 FIG S3 CU fimbrial ushers in non-*enterica* subspecies isolates detected on prophage or plasmid regions. Shown is the unrooted phylogeny of amino acid sequences for fimbrial ushers (with IPR annotation IPR000015) detected on prophage regions identified by Phaster (annotated as “phage regions” in the leaf label) or plasmid regions (annotated as “plasmid” in the leaf label) identified by Platon. Phylogeny was inferred using the LG+F+R5 substitution model, selected based on the BIC value. Branches are colored to reflect the fimbrial family of the usher. Download FIG S3, TIF file, 1.1 MB. Copyright © 2022 Cheng et al. 2022 Cheng et al. https://creativecommons.org/licenses/by/4.0/ This content is distributed under the terms of the Creative Commons Attribution 4.0 International license.

Overall, 12 ushers (StjB, BcfC, FimD, SddC/SdfC [i.e., sequences that shared similar %IDs to SddC and SdfC and could not be assigned to SddC or SdfC], StgC, SthB, PegC, PehC, StbC, StcC, SbbB, and StdB) were detected in multiple subspecies *enterica* clades and multiple non-*enterica* subspecies, supporting that most of these ushers were likely acquired prior to the divergence of the different S. enterica subspecies; SbaC was detected in multiple non-*enterica* subspecies but only in subspecies *enterica* clade B (Fig. 8A). An additional 7 ushers (LpfC, SdfC, SafC, SdgB, StaC, StfC, and SteB) were detected in one or more subspecies *enterica* clades and one non-*enterica* subspecies. While a single subspecies *diarizonae* isolate carried SafC, given the rarity of this usher among non-*enterica* subspecies isolates, this usher was most likely acquired by subspecies *enterica*, with the single subspecies *diarizonae* isolate having acquired SafC independently. Eight ushers (TcfC, StiC, SefC, StkC, SdhB, FaeC, PefC, and SafC/SefC [i.e., ushers sharing similar %IDs to both SafC and SefC; although classified as distinct ushers here, future studies will be necessary to confirm that these are distinct ushers and not just divergent sequences of SafC or SefC]) were detected only in subspecies *enterica* isolates, suggesting that these could have been acquired by subspecies *enterica*, especially since most are detected in multiple clades within this subspecies (Fig. 8A). SdiC, although present in isolates from multiple non-*enterica* subspecies isolates, was not detected in any subspecies *enterica* isolates, suggesting that this fimbria was likely lost prior to the divergence of subspecies *enterica* and *indica*. Finally, SddC (these sequences shared 100% sequence identity with the SddC reference and were therefore classified as SddC instead of SddC/SdfC) and SdcD were found only in subspecies *arizonae*. Altogether, these data suggest that the majority of fimbrial ushers were acquired prior to the divergence of subspecies *enterica*, although some ushers show patterns of loss by select phylogenetic clades.

### Similar numbers of hypothetically disrupted coding sequences annotated as having a fimbrial function are found among known host-adapted and broad-host-range isolates.

Previous research suggested that known host-adapted serovars exhibited degradation of genes in metabolic pathways, while broad-host-range generalists maintained these pathways (39). Given the proposed role of fimbriae in facilitating host adaptation (23, 24), we compared the numbers of coding sequences marked as “pseudogenes” (designated hypothetically disrupted coding sequences [HDCs] in this study) that were also annotated as having a function related to “fimbrial” biogenesis. While the overall median number of total HDCs per Salmonella genome was 139 (Fig. S4), the median number of HDCs annotated as being involved in fimbrial biogenesis was just 1 per genome, although this varied by clade (Fig. 8B). Isolates in subspecies *enterica* clade C had the highest (*n* = 3) median number of fimbrial HDCs per genome, while isolates in subspecies *enterica* clades A1 and D and isolates in subspecies *salamae* and *houtenae* had a median of 0 fimbrial HDCs per genome (Fig. 8B). Overall, these results suggest that the number of fimbrial HDCs is relatively low, regardless of the phylogenetic clade.

10.1128/msystems.00115-22.4 FIG S4 Comparison of the total numbers of hypothetically disrupted coding sequences (HDCs) in all Salmonella species isolates. HDCs were extracted from genome annotations provided using PGAP software. Download FIG S4, TIF file, 0.8 MB. Copyright © 2022 Cheng et al. 2022 Cheng et al. https://creativecommons.org/licenses/by/4.0/ This content is distributed under the terms of the Creative Commons Attribution 4.0 International license.

As the host range of most serovars is unknown, we next looked at specific serovars with known host associations. For isolates representing *S.* Typhi (host restricted to humans) and *S.* Choleraesuis (host adapted to pigs), the numbers of fimbrial HDCs were higher (5 and 4 fimbrial HDCs, respectively), but for *S.* Dublin (host adapted to cattle) and *S.* Paratyphi A (host restricted to humans), the numbers of fimbrial HDCs were identical (2 fimbrial HDCs) to the number of fimbrial HDCs in isolates representing *S.* Typhimurium and *S.* Infantis, both of which are considered to be broad-host-range serovars (Table S1). Interestingly, isolates in subspecies *arizonae* and *diarizonae*, which are often associated with reptiles (40), although they can be carried by warm-blooded animals (41), had higher median numbers of total HDCs than isolates in most subspecies *enterica* clades (Fig. S4) but had median numbers of fimbrial HDCs similar to those of the host-adapted subspecies *enterica* serovars (Table S1). Taken together, these results suggest that in contrast to what has been observed for the degradation of genes in metabolic pathways accompanying host adaptation, the number of fimbrial HDCs does not have an obvious correlation with host adaptation.

10.1128/msystems.00115-22.5 TABLE S1 Comparison of the numbers of total and fimbria-associated hypothetically disrupted coding sequences (HDCs) among select serovars with a known host association. Download Table S1, DOCX file, 0.02 MB. Copyright © 2022 Cheng et al. 2022 Cheng et al. https://creativecommons.org/licenses/by/4.0/ This content is distributed under the terms of the Creative Commons Attribution 4.0 International license.

## DISCUSSION

Salmonella represents one of the most successful bacterial pathogens, owing in part to its ability to colonize or infect an impressive repertoire of hosts, including mammals, amphibians, reptiles, birds, and fish (31, 42, 43). Although S. enterica serovars differ in the strategies that they employ to establish an infection, the ability to adhere to the gut epithelium is conserved in both typhoidal and nontyphoidal serovars. We identified few fimbrial ushers that represented divergent or potentially novel fimbriae, with most of these sequences being either associated with carriage on a plasmid or identified in isolates in subspecies *enterica* phylogenetic clades C and D. Furthermore, our analysis of Salmonella isolates representing S. bongori and non-*enterica* subspecies within S. enterica indicated that few fimbriae are unique to subspecies *enterica*, suggesting that many were acquired prior to the divergence of S. enterica subsp. *enterica*. Overall, our data revealed a strong phylogenetic relationship between the number and type of fimbriae encoded, suggesting that clade, rather than host, is associated with the presence of a given fimbria.

### Plasmids play an important role in the horizontal transfer of fimbrial ushers sharing lower sequence identity with reference ushers.

Mobile elements such as plasmids, prophages, and integrative conjugative elements have been previously associated with the mobilization and acquisition of (i) genes conferring antimicrobial (44) and metal (45) resistance, (ii) virulence factors (46–48), and (iii) O antigen modification enzymes (49, 50) in Salmonella. Previously, only the κ-fimbriae Pef and Fae were characterized as being predominantly associated with plasmids (36, 51, 52), although recent characterizations of some divergent plasmid-associated CU fimbriae have been described (29). In addition to confirming the presence of PefC and some FaeC ushers on plasmids, we also found evidence of the plasmid-mediated acquisition of α-fimbrial ushers (TcfC), γ-fimbrial ushers (SafC, SefC, and divergent γ-fimbrial ushers), and π-fimbrial ushers (SbbC and divergent π-fimbrial ushers), suggesting that plasmids likely play an important role in mediating the transfer and acquisition of multiple CU fimbriae. Conversely, just 22 fimbrial ushers (14 FimD, 3 StbC, 2 StdB, 2 SafC, and 1 κ-fimbrial usher) were associated with genomic regions characterized as prophages, with all but one prophage being characterized as “incomplete” or “questionable” (also known as defective), suggesting that the prophage-mediated acquisition of CU fimbrial gene clusters may have occurred previously in the evolution of Salmonella, such as for the acquisition of FimD, which was found on prophage sequences in some isolates in subspecies *enterica*, *arizonae*, and VII, but is likely not a primary means of acquiring fimbrial gene clusters. Conversely, a number of the fimbrial ushers that mapped to contigs associated with plasmids shared lower amino acid sequence identity with known reference sequences, suggesting that plasmids likely serve as mediators of the acquisition of fimbriae from other genera, as genera within the *Enterobacteriaceae* family are known to share plasmids (53, 54), with horizontal transfer occurring in the intestinal tract (55). Indeed, some of the divergent fimbriae within the γ-fimbrial family identified here shared high sequence identity with fimbrial ushers from *Citrobacter* spp. and Escherichia spp. In our analysis, most of the FaeC ushers identified were not associated with contigs assigned to plasmids. This result was unexpected as the Fae fimbrial gene cluster has previously been associated with plasmid sequences in Salmonella (36). As plasmid sequences are notoriously challenging to assemble from short-read sequence data (56), it is possible that the FaeC sequences identified here were unable to be assigned to plasmid contigs, although all 5 PefC sequences identified in this study were associated with contigs assigned to plasmids (likely the Salmonella virulence plasmid). Regardless, our findings suggest that plasmids represent important vectors for facilitating the horizontal transfer of fimbriae among S. enterica serovars, even though our data may underestimate the overall proportion of CU fimbrial genes carried on plasmids.

### CU fimbrial ushers in subspecies *enterica* clade C and D serovars often represent divergent sequences.

Serovars within subspecies *enterica* phylogenetic clades A1, A2, B and section Typhi account for the majority of clinical salmonellosis cases in humans and animals (8). Our initial analyses also included isolates representative of all known serovars (8) from clades D (Salmonella serovars Montaigu, Mendoza, Westminster, and Brazzaville) and C (Salmonella serovars Maricopa, Sanjuan, Denver, Weslaco, and Newmexico), which are rarely isolated from human clinical cases (6). Overall, we observed that many of the fimbrial ushers from isolates in clades C and D and Salmonella serovars Lattenkamp and Poano, which could not be assigned to a major phylogenetic group, shared high sequence identity with the reference usher sequences. This, combined with the observed presence of many homologous fimbrial ushers in isolates from non-*enterica* subspecies, suggests that many of the fimbrial ushers were likely acquired by a common ancestor prior to the divergence of subspecies *enterica*. However, we also observed that some of the fimbrial usher sequences from clades C and D and Salmonella serovars Lattenkamp and Poano represented divergent sequences that shared low amino acid identity with reference sequences but high amino acid sequence identity with sequences from other isolates belonging to clades C and D. For example, clade D serovars encoded divergent sequences of StjB (β-fimbriae) as well as the π-fimbrial ushers StfC and SteB, while *S.* Lattenkamp and *S.* Poano encoded divergent sequences of StjB as well as γ_3_ SafC/SefC (i.e., these fimbriae shared similar %IDs with the SafC and SefC references). Furthermore, clade C serovars encoded divergent sequences of FaeC, SbbC, and γ-, π-, and κ-fimbrial ushers that shared low identity with reference sequences. As these divergent sequences could have been generated by recombination events, future in-depth analyses of the evolution of fimbrial families and individual fimbrial genes, including efforts to infer the potential role of recombination in generating the observed sequence diversity of CU fimbrial ushers, will be important.

### Only 8 CU fimbrial ushers are unique to subspecies *enterica* serovars.

While the initial set of 242 genome sequences used to study the diversity of CU fimbriae represented those serovars commonly associated with human and animal hosts, all of these genomes belong to S. enterica subsp. *enterica*, consistent with the fact that this group is responsible for the vast majority of Salmonella infections in humans and warm-blooded animals (4). We thus extended our efforts to include the characterization of fimbrial ushers in a broader range of Salmonella isolates, including multiple subspecies, complementing previous studies that characterized fewer isolates (26, 30) or did not include all known fimbrial gene clusters (8). Our data suggest that (i) most fimbrial ushers are found throughout multiple Salmonella phylogenetic lineages, suggesting their acquisition prior to the divergence of subspecies *enterica*, and (ii) many of the recently described fimbriae (e.g., Peg, Peh, Sba, Sbb, and Sdh) (26) are more widely distributed than previously thought. With the exception of the TcfC, StiC, SefC, SafC/SefC (i.e., fimbriae sharing similar percent identities with both SafC and SefC), StkC, SdhB, PefC, and FaeC fimbrial ushers, all other ushers are encoded by at least some isolates of S. bongori and/or the non*-enterica* subspecies; this most likely suggests that Tcf, Sti, Sef, Saf/Sef, Stk, Sdh, Pef, and Fae were acquired horizontally by subspecies *enterica*, as 7 of these ushers (i.e., PefC was found only in clade A2 isolates) are present in multiple clades within this subspecies. SdcD (σ-fimbria) was the only other fimbrial usher that was detected in only a single subspecies (subspecies *arizonae*), suggesting acquisition in this lineage. While SddC was also identified only in subspecies *arizonae* isolates, it should be noted that SddC and SdfC (which is present in other subspecies) share 93% percent identity, suggesting that they are homologs (and therefore may represent the same usher/fimbria); a SddC homolog was thus likely present in the common ancestor of the different Salmonella subspecies and diversified in subspecies *arizonae*.

### Isolates representing serovars with known host adaptations do not possess higher numbers of fimbrial HDCs.

Pseudogenization, or the acquisition of mutations within a gene that affect the function of the resulting gene product, has been documented previously as a signature accompanying host adaptation of Salmonella, especially among genes involved in metabolic pathways (13, 39, 57). For example, Nuccio and Bäumler showed that host-adapted/restricted isolates (designated extraintestinal pathovars in that study) had higher numbers of HDCs among genes involved in the regulation of fatty acid degradation, carbohydrate catabolism, and anaerobic respiration (39) than host generalists (designated gastrointestinal pathovars). In contrast, we found that while the number of HDCs was higher among non-*enterica* subspecies isolates, the number of HDCs annotated as being involved in a fimbrial pathway was relatively low overall, with most clades encoding a median of 1 to 2 fimbrial HDCs. This may suggest that other evolutionary processes, such as the positive selection of certain alleles of tip adhesins with a higher affinity for binding specific host cell epitopes (23, 24), may play a more important role in fimbria evolution and host adaptation than the potential loss of function of genes in the fimbrial biogenesis pathway. However, additional analyses examining fimbrial HDCs among multiple isolates within a given serovar will be necessary for asserting this, as some studies suggest that adaptation of clades within a serovar may occur (16). Finally, as some fimbrial gene clusters can include multiple chaperones, and, therefore, the loss of one chaperone may not necessarily result in the loss of the fimbriae, functional characterization of different fimbriae will also be necessary to fully understand the implications of the fimbrial HDCs identified here and their effect on fimbria function as well as any potential role in host adaptation.

### The high sequence similarity shared between some fimbrial ushers highlights the need for standardized nomenclature and criteria for classifying fimbrial ushers.

Genomic characterization of nontyphoidal Salmonella serovar Typhimurium (33) and typhoidal Salmonella serovar Typhi (27) led to the identification of roughly half of the fimbriae described for Salmonella. Additional characterizations of S. bongori (31) and other S. enterica subspecies (26, 30) further expanded the Salmonella fimbriome, with the discovery of additional, “novel” fimbrial gene clusters. In the absence of standardized criteria, the presence of genes that share lower identity with reference sequences from the model Salmonella serovars Typhimurium (subspecies *enterica* clade A2) and Typhi (subspecies *enterica* section Typhi) may have led to the description of novel fimbriae that represented ancestral or divergent sequences of previously described fimbrial ushers. Indeed, our initial attempts to classify fimbrial ushers based on percent identity shared with reference sequences proved challenging, as a uniform cutoff (based on percent identity) could not be established. For example, PehC shares 96% amino acid identity with StcC and 89% identity with PegC. Similarly, we found that (i) SdgB shares high percent amino acid identity with SdhB (89%) and StaC (96%), (ii) SdhB shares 90% identity with StaC, (iii) SddC and SdfC share 93% identity, and (iv) SbaC and SdgB share 86% identity. Previously, work by Yue and colleagues showed that *pegABCDE*, *sdjABCD_yhoN* (associated with subspecies *diarizonae* [Sdj could not be included in our data set because a reliable reference sequence could not be found]), *pehABCDE*, and *stcABCD_yhoN* form a monophyletic clade, suggesting that these fimbriae share an ancestor (26). Yue and colleagues also noted that (i) Sdd and Sdf, (ii) Sba and Sdg, and (iii) Peg and Peh fimbrial gene clusters show synteny with regard to their genomic location (26), further supporting that these three pairs of fimbriae likely represent the same fimbriae, even though they are referred to by different names. These specific findings as well as our study point to a need for a standardized approach to the nomenclature of fimbriae and fimbrial families that is based on (i) a defined set of reference sequences (that are curated and updated); (ii) phylogenetic analysis of multiple genes in the fimbrial gene cluster, including ushers (as was done in this study) as well as chaperones, minor and major structural subunits, and transcriptional regulators; (iii) additional and in-depth evolutionary analyses, including analyses to understand the role of homologous recombination in generating diversity in different fimbrial genes; and (iv) functional characterizations assessing the expression and biological role and function of different fimbriae *in vitro*/*in vivo*. While these additional steps will require considerable computational and experimental analyses, they will be important for defining and understanding the role of these fimbriae in Salmonella serovars as well as in other Gram-negative pathogens, as many genomic investigations often include these virulence factors in characterizations (58–61).

Overall, the broad diversity of CU fimbriae identified in Salmonella spp., as well as across Gram-negative species, suggests that these organelles play an important role in mediating interactions between bacteria and their environments. E. coli has been shown to encode a similar number of distinct fimbriae (38 fimbrial gene clusters [38]), suggesting that the diversification of fimbriae may represent an important evolutionary trait for Gram-negative bacteria in general. Given that many fimbrial gene clusters have homologs in multiple Gram-negative bacterial species (18), understanding the roles that these fimbriae play in one species will likely provide functional insights into the role of fimbriae in mediating interactions of other Gram-negative species as well.

## MATERIALS AND METHODS

### Selection of serovars and representative isolates.

S. enterica subsp. *enterica* serovars were included based on their (i) prevalence among reported cases of human clinical infections from 2006 to 2016 in the United States (i.e., serovars having a median number of cases per year of ≥1 were included) (6), (ii) prevalence among nonhuman clinical and nonhuman nonclinical reported cases in 2012 (32) (i.e., serovars having a median of ≥5 cases across all nonhuman sources), or (iii) representation of a rare or novel clade of subspecies *enterica* based on previous studies (i.e., clade C and D serovars) (8). If the serovar contained known poly- or paraphyletic clades (i.e., here, polyphyletic serovar is used to signify two or more clades of isolates with an identical antigenic formula that do not share a most recent common ancestor [MRCA] with the same antigenic formula, while paraphyletic serovar is used to signify a clade of multiple isolates with an identical antigenic formula that share an MRCA with isolates that have a different antigenic formula), isolates representing each clade were selected (1 isolate per poly- or paraphyletic clade).

One isolate was selected to represent each serovar or clade in the case of poly- or paraphyletic serovars. Isolates were selected to represent the most prevalent single nucleotide polymorphism (SNP) cluster for that serovar in the NCBI pathogen detection database (https://www.ncbi.nlm.nih.gov/pathogens/). Using data available as of 13 January 2021, furthermore, among all isolates in a given cluster, we selected a genome that (i) was reported as being sequenced on an Illumina platform, (ii) had the highest *N*_50_ value, and (iii) had a genome size ranging from 4.5 to 5.7 Mbp. For poly- and paraphyletic serovars, isolates were selected from previously published studies for which raw reads were publicly available. Using this approach, a total of 242 genomes representing isolates from 217 S. enterica subsp. *enterica* serovars were included in our analyses (see Data Set S2 in the supplemental material for assembly and serovar information for selected isolates).

10.1128/msystems.00115-22.7 DATA SET S2 Isolate assembly metadata and *in silico* serotype prediction results. Download Data Set S2, XLSX file, 0.06 MB. Copyright © 2022 Cheng et al. 2022 Cheng et al. https://creativecommons.org/licenses/by/4.0/ This content is distributed under the terms of the Creative Commons Attribution 4.0 International license.

Initially, all available S. bongori assemblies available in the NCBI database (*n* = 32; accessed 15 July 2021) were downloaded. Because roughly half of these isolates represented 66:z_41_:– (*n* = 11 isolates) and 48:z_35_:– (*n* = 7 isolates) serotypes, and both formed monophyletic clades, a single isolate was selected to represent each of these two serotypes. Therefore, a total of 13 S. bongori isolates were selected for characterization (Data Set S2). Representative assemblies for the remaining S. enterica subspecies were selected based on the number of isolates in each NCBI pathogen detection database SNP cluster (accessed 15 July 2021). Assemblies with the highest *N*_50_ values from each of the top 5 most populous SNP clusters representing unique serotypes were selected for each subspecies (i.e., II, IIIa, IIIb, IV, and VI); for subspecies VII, due to the small number of assemblies available, selecting unique serotypes was not possible, and therefore, 4 of the 5 selected assemblies represent the same serotype.

### Genome assembly and annotation.

Assemblies and annotated genomes were downloaded from the NCBI database. Genomes for all subspecies *enterica* isolates were assembled with SKESA version 2.2, and genome annotations were downloaded (all annotations were performed using the NCBI PGAP pipeline [see Data Set S2 for specific versions used for each assembly]). For two isolates, assembled genomes were not available online. For these isolates, raw reads were assembled with SKESA and annotated with PGAP using RAPT version 0.2.0. Serotype information for assembled and annotated genomes was confirmed using the Salmonella *in silico* typing resource (SISTR) command-line tool, version 1.0.2 (62).

### S. enterica subsp. *enterica* clade designation based on core single nucleotide polymorphisms.

Core SNPs were identified and aligned with kSNP3 version 3.1 using a kmer size of 19 (suggested by the output from kchooser [63]). A phylogeny based on core SNPs was inferred using IQ-TREE v.2.0.7 with the substitution model GTR+G+ASC (General Time Reversible with gamma distribution and ascertainment bias correction), with 1,000 ultrafast (UF) bootstrap repetitions (64, 65). S. enterica subsp. *enterica* phylogenetic clades were assigned in accordance with those designated previously (8). Note that in the phylogeny described here, clades A1 and A2 are both paraphyletic; isolates representing Salmonella serovars (i) Takoradi and Goldcoast (clade A1) and (ii) Virchow and Colindale (clade A2) were assigned a clade designation based on the findings of Worley and colleagues (8) due to the low bootstrap support obtained in our phylogeny. Finally, for two isolates representing Salmonella serovars Lattenkamp and Poano, which represented distinct branches in the phylogeny and which were not included in the analyses of Worley and colleagues (8), the isolates were treated as potentially novel clades and, therefore, were not assigned to an existing phylogenetic clade.

### Usher protein family assignment and putative usher identification.

InterProScan version 5.44-79.0 was used to assign InterPro superfamilies for coding sequences in annotated genomes. The resulting output files were queried, and the amino acid sequences for all coding sequences annotated as IPR000015 (representing the outer membrane usher protein family) were extracted for further analysis; the utility of this approach was confirmed for representative amino acid sequences for 29 previously identified Salmonella fimbrial usher proteins. For the Typhi colonization factor (Tcf) fimbrial usher protein, IPR035224 (representing the TcfC usher-like barrel domain) was used, as TcfC is not included in the IPR000015 family. Sequences were included in phylogenetic analyses if they included at least 711 amino acid residues (representing 90% of the length of SdcD, which was the shortest reference CU fimbrial usher sequence); sequences shorter than this threshold were considered partial sequences and were removed from the data set (*n* = 31 sequences). Amino acid sequences corresponding to fimbrial usher proteins with IPR000015/IPR035224 and representative sequences of previously identified fimbrial ushers (i.e., sequences corresponding to known fimbrial usher proteins extracted from WGS data for *S.* Typhimurium LT2 or S. Typhi CT18 or from the isolate in which the fimbrial gene cluster was identified [see Table 2 for sequence accession number information]) were aligned with MAFFT version 7.453, a phylogeny was inferred for all usher proteins using IQ-TREE v.2.0.7 (64) with the –m MFP option (66) to identify the best-fitting amino acid substitution model, and clade confidence was assessed using 1,000 ultrafast bootstrap repetitions (65). Fimbrial ushers were then assigned to a fimbrial usher family (α, β, γ, κ, π, or σ) based on their sharing an MRCA with a known fimbrial usher protein representative of that family. After assigning each fimbrial usher protein to a family (i.e., α, β, γ, κ, π, or σ), proteins were realigned with representative members of the fimbrial protein family using MAFFT, and a phylogeny was inferred as described above with IQ-TREE software. All trees were visualized and edited in iTOL (67). Percent identities shared between individual ushers and reference ushers were calculated in Geneious (Biomatters, Inc., San Diego, CA, USA) using the BLOSUM-62 matrix. Ushers were assigned an identification based on the percent identity shared between the usher and the reference (note that a universal cutoff could not be applied due to the sequence diversity of the ushers and references) and the tree topology; identities of all proteins are listed in Data Set S3.

**TABLE 2:** Reference fimbrial ushers used for identifying usher proteins annotated as IPR000015 or IPR035224

CU fimbria	Class	Usher gene	Strain	NCBI accession no.
Bcf	γ_1_	*bcfC*	*S.* Typhi strain CT18	NC_003198.1
Fae	κ	*faeC*	*S.* Poona strain NCTC4840	LS483489.1
Fim	γ_1_	*fimD*	*S.* Typhi strain CT18	NC_003198.1
Lpf	γ_1_	*lpfC*	*S.* Typhimurium strain LT2	AE006468.2
Mrk	γ_4_	*mrkC*	*S.* Montevideo strain CDC2012K-1544	CP017977.1
Pef	κ	*pefC*	*S.* Typhimurium strain LT2	NC_003277.2
Peg	γ_4_	*pegC*	*S.* Enteritidis strain SE95	CP050716.1
Peh	γ_4_	*pehC*	*S.* Weltevreden strain HI_N05-537	NZ_ABFF01000001.1
Saf	γ_3_	*safC*	*S.* Typhimurium strain LT2	AE006468.2
Sba	γ_4_	*sbaC*	S. bongori strain NCTC12419	NC_015761.1
Sbb	π	*sbbB*	S. bongori NCTC12419	NC_015761.1
Sdc	σ	*sdcC*	S. enterica subsp. *diarizonae* 62:z4,z23:–	NC_010067.1
Sdd	γ_1_	*sddC*	S. enterica subsp. *diarizonae* 62:z4,z23:–	NC_010067.1
Sdf	γ_1_	*sdfC*	*S.* Schwarzengrund strain CVM19633	NC_011094.1
Sdg	γ_4_	*sdgB*	*S.* Schwarzengrund strain CVM19633	NC_011094.1
Sdh	γ_4_	*sdhB*	*S.* Agona strain SL483	CP001138.1
Sdi	γ_4_	*sdiC*	S. enterica subsp. VII strain SARC16	FN298495.1
Sef	γ_3_	*sefC*	*S.* Typhi strain CT18	NC_003198.1
Sta	γ_4_	*staC*	*S.* Typhi strain CT18	NC_003198.1
Stb	γ_4_	*stbC*	*S.* Typhimurium strain LT2	AE006468.2
Stc	γ_4_	*stcC*	*S.* Typhimurium strain LT2	AE006468.2
Std	π	*stdB*	*S.* Typhimurium strain LT2	AE006468.2
Ste	π	*steB*	*S.* Typhi strain CT18	NC_003198.1
Stf	π	*stfC*	*S.* Typhimurium strain LT2	AE006468.2
Stg	γ_1_	*stgC*	*S.* Typhi strain CT18	NC_003198.1
Sth	γ_1_	*sthB*	*S.* Typhimurium strain LT2	AE006468.2
Sti	γ_1_	*stiC*	*S.* Typhimurium strain LT2	AE006468.2
Stj	β	*stjB*	*S.* Typhimurium strain LT2	AE006468.2
Stk	γ_4_	*stkC*	*S.* Heidelberg CFSAN002069	NC_021812.1
Tcf	α	*tcfC*	*S.* Typhi strain CT18	NC_003198.1

### Assessment of plasmid- and prophage-associated fimbrial usher proteins.

To determine if the usher protein mapped to sequences predicted to be from plasmids or prophages, we used Platon version 1.3.1 (68) or Phaster (69), respectively. Sequences identified as plasmids or prophages were then annotated with Prokka version 1.14.5 (70) to define coding sequences, followed by annotation with InterProScan version 5.44-79.0 to identify IPR000015 and IPR035224 proteins, as described above.

### Detection of hypothetically disrupted coding sequences.

Hypothetically disrupted coding sequences were identified among annotated assemblies downloaded from the NCBI database (see Data Set S2 for assembly accession number information). The GenBank to Fasta python script (https://rocaplab.ocean.washington.edu/tools/genbank_to_fasta/) was used to convert data into fasta format, followed by the use of a custom python script (71) to parse coding sequences based on PGAP annotation. A search for “fimbrial” in the header was used to extract HDCs with the functional annotation “fimbrial” (note that this method may also identify nonusher fimbrial genes and some non-CU fimbrial genes).

### Data availability.

All sequence data are publicly available in the NCBI database. All data associated with this article are included in the supplemental material (Data Sets S1 to S3).
