# Supplementary figures and images for: The Number and Type of Chaperone-Usher Fimbriae Reflect Phylogenetic Clade Rather than Host Range in Salmonella
Source: mSystems. 2022 Apr 25;7(3):e00115-22. doi: 10.1128/msystems.00115-22 (PMC9238391; doi:10.1128/msystems.00115-22)

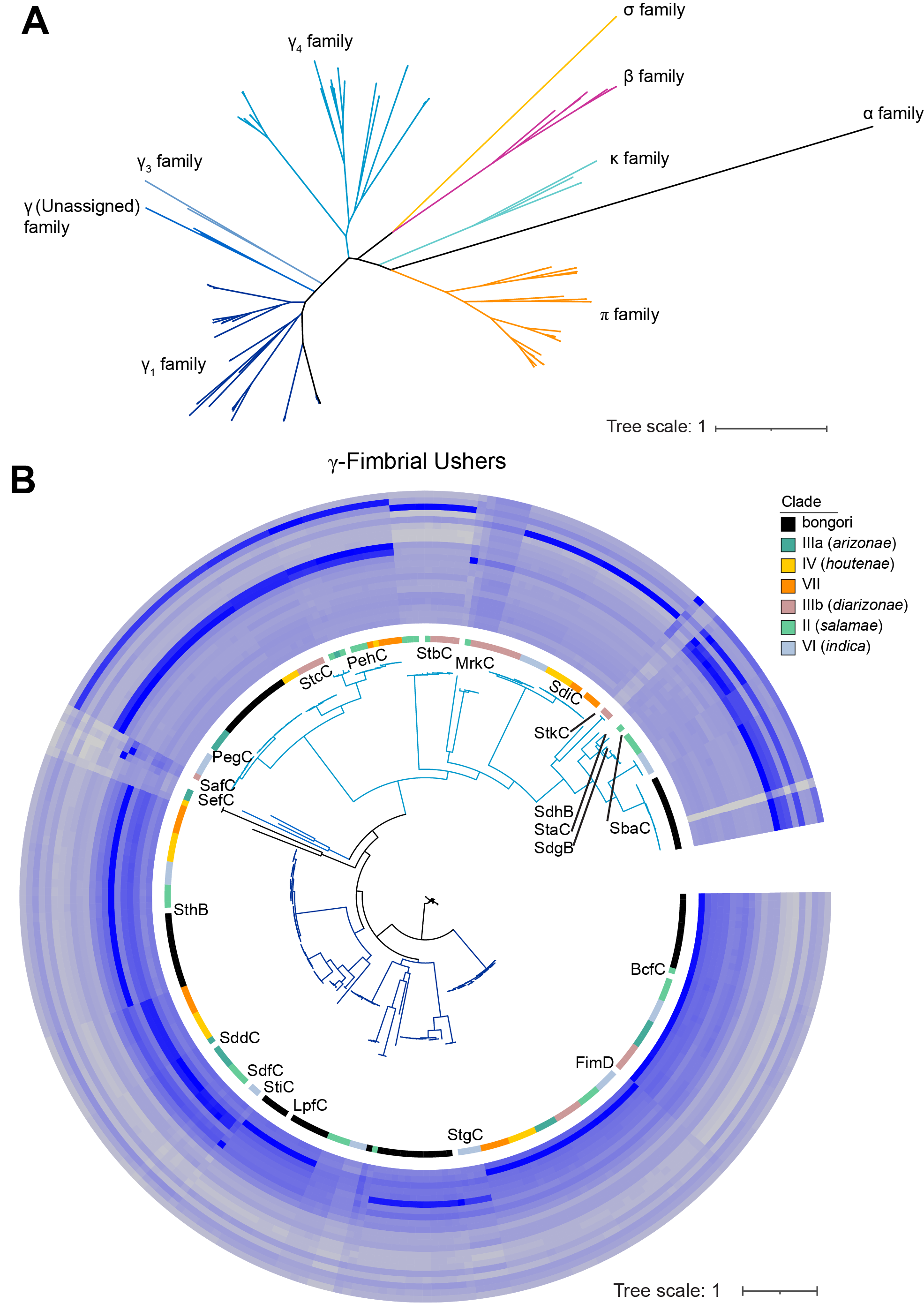

Supplement: FIG S1 [file msystems.00115-22-s0001.tif]

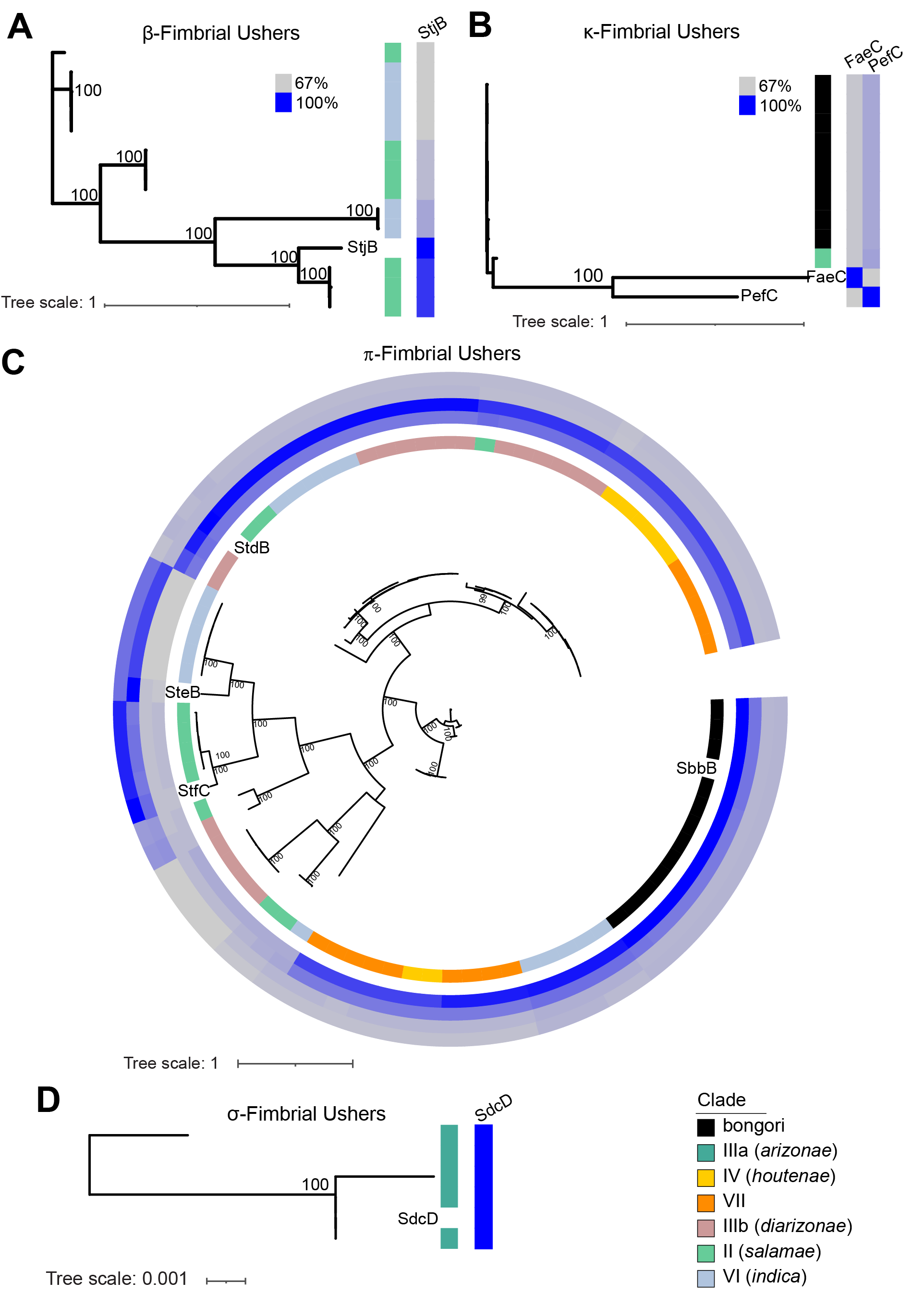

Supplement: FIG S2 [file msystems.00115-22-s0002.tif]

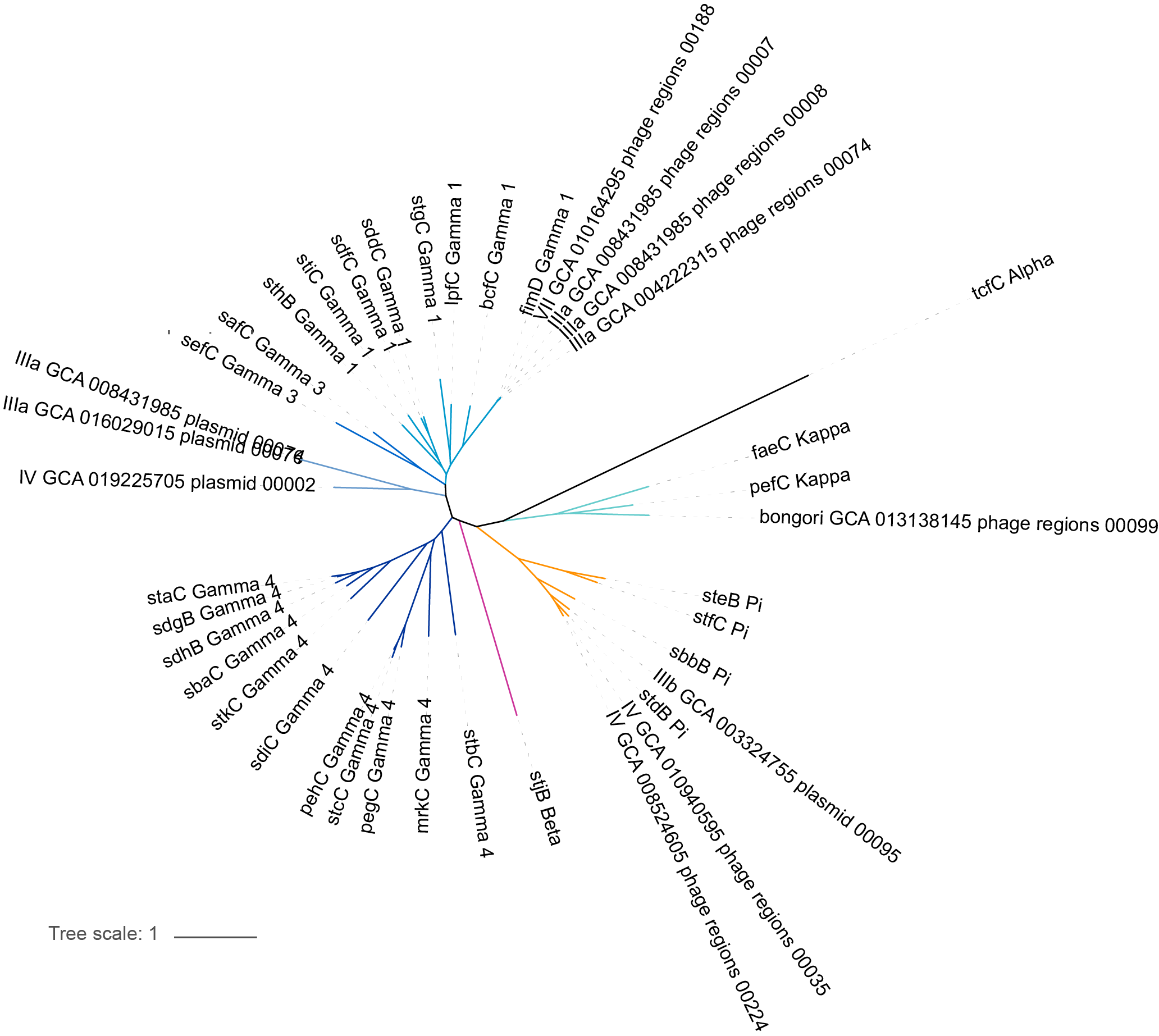

Supplement: FIG S3 [file msystems.00115-22-s0003.tif]

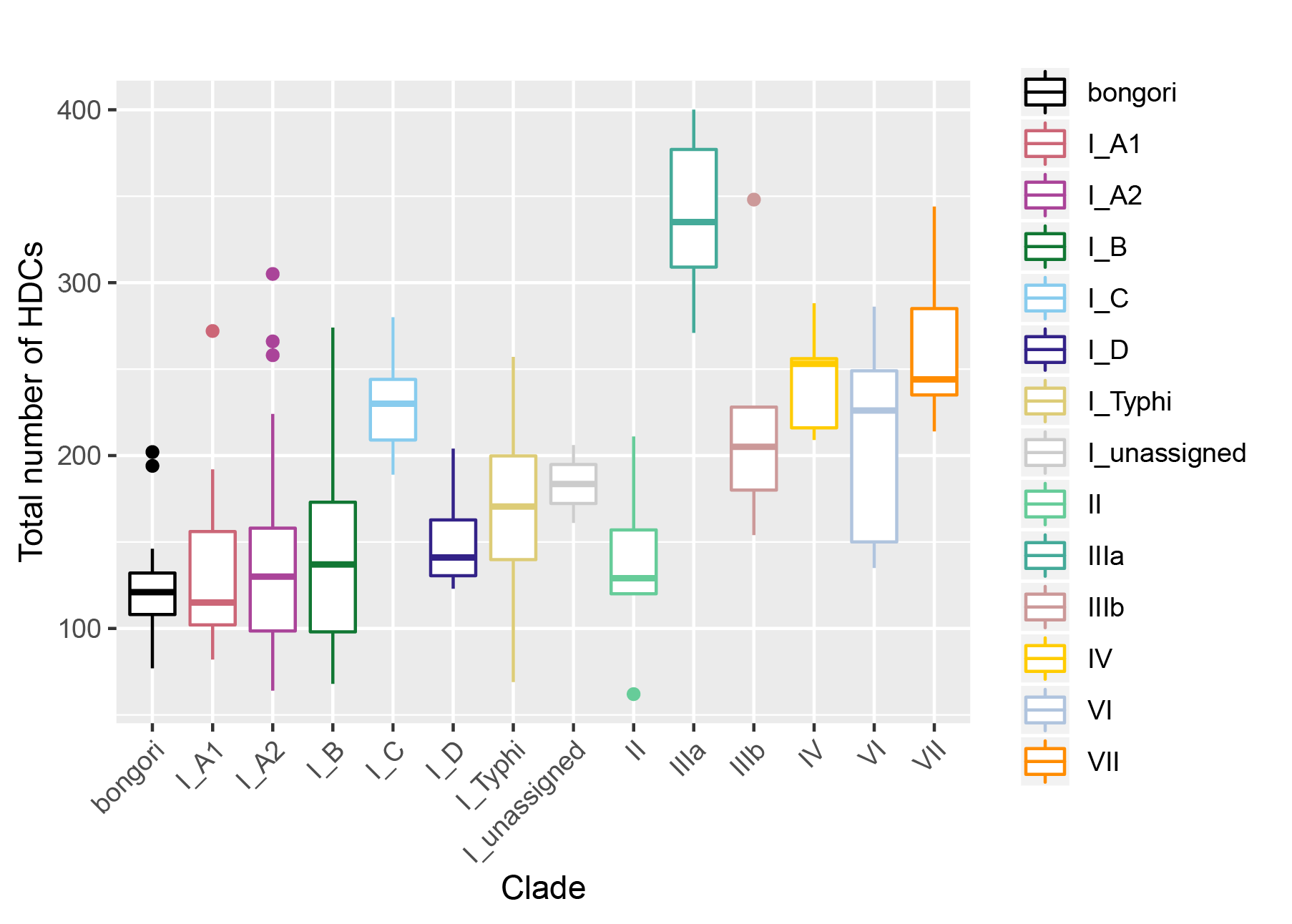

Supplement: FIG S4 [file msystems.00115-22-s0004.tif]
