# Supplementary material for: The Number and Type of Chaperone-Usher Fimbriae Reflect Phylogenetic Clade Rather than Host Range in Salmonella
Source: mSystems. 2022 Apr 25;7(3):e00115-22. doi: 10.1128/msystems.00115-22 (PMC9238391; doi:10.1128/msystems.00115-22)
Supplement: TABLE S1 [file msystems.00115-22-s0005.docx]

| **Subspecies** | **Serovar** | **Assembly** | **# total HDCs^a^** | **# fimbrial HDCs^b^** | **Notes^c^** |
| --- | --- | --- | --- | --- | --- |
| I | Paratyphi A | GCA_005604015 | 222 | 2 | extra intestinal, host-restricted to humans |
| I | Typhi | GCA_010528315 | 257 | 5 | extra intestinal, host-restricted to humans |
| I | Dublin | GCA_008431285 | 258 | 2 | extra intestinal, host-adapted to cows |
| I | Choleraesuis | GCA_007748275 | 305 | 4 | extra intestinal, host-adapted to pigs |
| I | Heidelberg | GCA_007742375 | 102 | 1 | Gastrointestinal |
| I | Paratyphi B | GCA_008466925 | 175 | 1 | Gastrointestinal |
| I | Infantis | GCA_009122245 | 175 | 2 | Gastrointestinal |
| I | Typhimurium | GCA_008432425 | 184 | 2 | Gastrointestinal |
| IIIa | IIIa 13,23:z4,z23,[z32]:- | GCA_016029015 | 271 | 2 | Reptile-associated |
| IIIa | IIIa 48:z4:z24:- | GCA_007969565 | 309 | 1 | Reptile-associated |
| IIIa | IIIa 41:z4,z23:- | GCA_009087135 | 335 | 1 | Reptile-associated |
| IIIa | IIIa 1,13,23:g,z51:- | GCA_008431985 | 377 | 2 | Reptile-associated |
| IIIa | IIIa 18:z4,z23:- | GCA_004222315 | 400 | 2 | Reptile-associated |
| IIIb | IIIb 47:k:z35 | GCA_017337585 | 154 | 1 | Reptile-associated |
| IIIb | IIIb 61:z52:z53 | GCA_010833345 | 180 | 1 | Reptile-associated |
| IIIb | IIIb 61:k:1,5,[7] | GCA_013328855 | 205 | 3 | Reptile-associated |
| IIIb | IIIb 50:k:z | GCA_005803545 | 228 | 1 | Reptile-associated |
| IIIb | IIIb 48:i:z | GCA_003324755 | 348 | 1 | Reptile-associated |

^a^total number of coding sequences classified as hypothetically disrupted coding sequences due to the presence of a premature stop codon

^b^total number of coding sequences classified as hypothetically disrupted coding sequences that were annotated as ‘fimbrial’ by PGAP annotation software

^c^For subspecies I serovars, we adopted the nomenclature used by Nuccio and Baumler (1).

**References**

1. Nuccio S-P, Bäumler AJ. 2014. Comparative analysis of *Salmonella* genomes identifies a metabolic network for escalating growth in the inflamed gut. mBio 5:e00929.
